# New tandem Ugi/intramolecular Diels–Alder reaction based on vinylfuran and 1,3-butadienylfuran derivatives

**DOI:** 10.3762/bjoc.21.31

**Published:** 2025-02-26

**Authors:** Yuriy I Horak, Roman Z Lytvyn, Andrii R Vakhula, Yuriy V Homza, Nazariy T Pokhodylo, Mykola D Obushak

**Affiliations:** 1 Department of Organic Chemistry, Ivan Franko National University of Lviv, 6 Kyryla i Mefodiya St., Lviv 79005, Ukraine

**Keywords:** 1,3-butadienylfuran, furo[2,3-*f*]isoindole, intramolecular Diels–Alder reaction, isoindole, one-pot, Ugi reaction, vinylfuran

## Abstract

A new tandem sequence involving the Ugi reaction and Diels–Alder [4 + 2] cycloaddition based on vinylfuran and 1,3-butadienylfuran derivatives was designed and studied. It was found that in the case of 3-(furan-2-yl)acrylaldehyde, a one-pot Ugi reaction and intramolecular Diels–Alder vinylarene (IMDAV) reaction leads to the formation of the insufficiently studied furo[2,3-*f*]isoindole derivatives. Ugi adducts formed from (*E*)-3-(furan-2-yl)acrylaldehyde, maleic acid monoanilide, isonitrile, and an amine spontaneously underwent the IMDAV reaction with a high level of stereoselectivity, leading to single pairs of enantiomers of 4,4a,5,6,7,7a-hexahydro-3a*H*-furo[2,3-*f*]isoindole core in excellent yields. Under the same conditions, the (2*E*,4*E*)-5-(furan-2-yl)penta-2,4-dienal gives an Ugi adduct that undergoes the IMDA reaction without involving the furan core. The cycloaddition leads to the formation of 2,3,3a,4,5,7a-hexahydro-1*H*-isoindoles in high yields. The studied tandem Ugi and intramolecular Diels–Alder reactions allow high substituent variation in the named isoindoles.

## Introduction

Energy-saving and environmentally friendly synthetic strategies, especially one-pot multicomponent and tandem reactions, are key to modern organic and medicinal chemistry [1–7] and have proven to be successful in generating diverse heterocyclic scaffolds, as highlighted in a recent book [8]. Multicomponent reactions, for instance, have contributed to high-throughput screening for drug discovery, facilitating the identification of new therapeutic compounds. [9–10]. A notable example is ivosidenib, approved in 2018, which was synthesized using the Ugi reaction to target mutated IDH1 in acute myeloid leukemia [11–13]. Advancements in one-pot syntheses, like combining the Ugi reaction with other methods [14–18], for example, the Huisgen cycloaddition, have led to the creation of unique [1,2,3]triazolo[1,5-*a*]pyrazine derivatives [19]. Tandem reactions are particularly valued for their atom and step-economy, making them promising for future commercial applications.

Partly hydrogenated isoindoles and their derivatives exhibit broad biological activities and are considered privileged motifs in medicinal chemistry [20–21]. These compounds, when condensed with aromatics or heterocycles, form heterolignans, which are synthetic derivatives of naturally occurring lignans [22–25]. This has gained significant attention in drug discovery [26].

**Figure 1:**
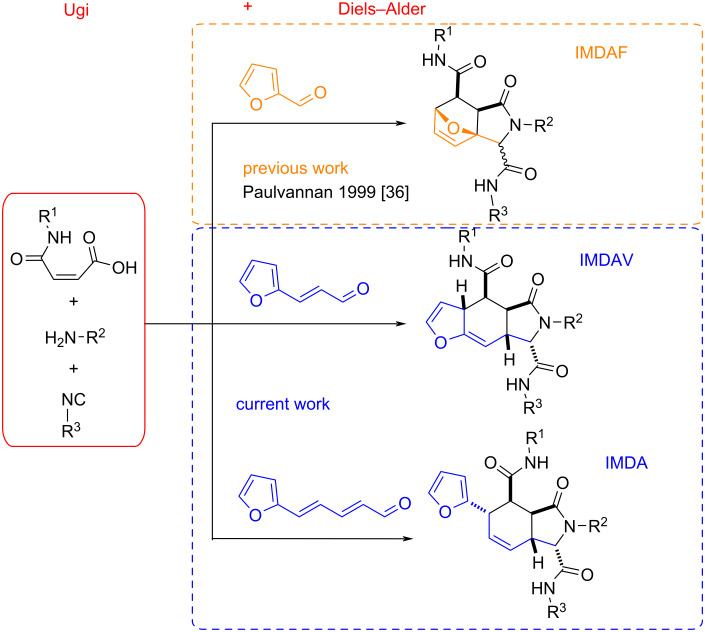
State of the art of Ugi/Diels–Alder reaction based on furan.

Several synthetic routes, such as tandem Pummerer/Diels–Alder or Wittig/Diels–Alder approaches, have been developed for heterolignan construction [27–28]. For instance, the intramolecular Diels–Alder reactions of vinylarenes (IMDAV) strategy [29–30] has been used to synthesize annulated isoindoles, including thieno[2,3-*f*]isoindoles [31–32] and furo[2,3-*f*]isoindoles [33–34], from thienyl- or furylallylamines and unsaturated acid derivatives. Also, benzoisoindoles have been synthesized via the pseudo-four-component reaction of 3-phenylallylamines with maleic anhydride [35]. Finally, furfural was utilized in the Ugi reactions with unsaturated acids leading to the intramolecular Diels–Alder furan (IMDAF) reaction (Figure 1) [36].

In the present work, the new tandem sequence involving an Ugi reaction and Diels–Alder [4 + 2] cycloaddition based on vinylfuran, 1,3-butadienylfuran derivatives was designed and studied.

## Results and Discussion

**Scheme 1:**
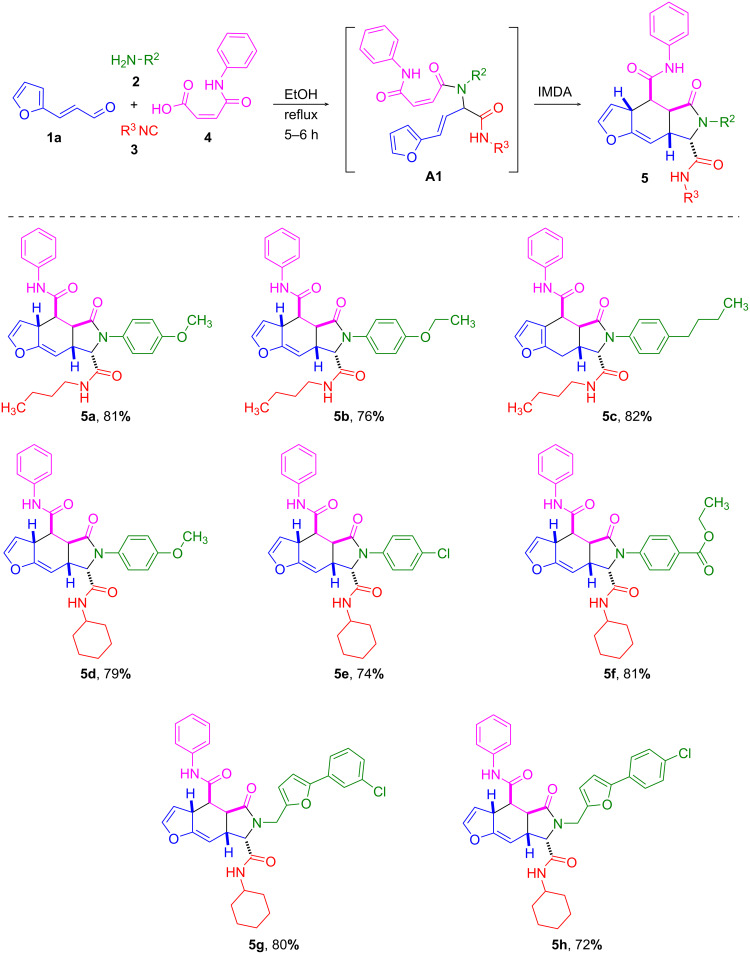
Preparation of 4,4a,5,6,7,7a-hexahydro-3a*H*-furo[2,3-*f*]isoindoles via Ugi/IMDAV tandem reaction.

To carry out an intramolecular Diels–Alder reaction, we tested (*E*)-3-(furan-2-yl)acrylaldehyde (**1a**) in the Ugi reaction with a maleic acid to form adducts containing both, diene and dienophilic fragments. It was found that aldehyde **1a** reacted with amines **2**, isonitriles **3**, and maleic acid monoanilide (**4a**) to form an adduct that spontaneously underwent a [4 + 2] cycloaddition giving furoisoindoles **5a**–**h** under the Ugi reaction conditions (Scheme 1). A furan double bond and an exocyclic double bond, forming the diene, enter the intramolecular cyclization, and a residue formed by the monoanilide of maleic acid acts as a dienophile. Notably, non-cyclized Ugi adducts **A1** were not found in the products. The products were identified via NMR as 4,4a,5,6,7,7a-hexahydro-3a*H*-furo[2,3-*f*]isoindoles.

The structures of compounds **5** were confirmed by NMR spectroscopy data (for details and discussion see Supporting Information File 1). The NMR data for the obtained furoindole skeleton signals and spin-coupling constants agree with our previous results on tandem acylation/Diels–Alder reactions [31–33] and lead us to conclude that the final Diels–Alder reaction products are formed as the *exo*-adducts. Moreover, since we do not observe other stereoisomers we speculate that the Ugi and Diels–Alder reactions occur under a coordinated mechanism.

**Scheme 2:**
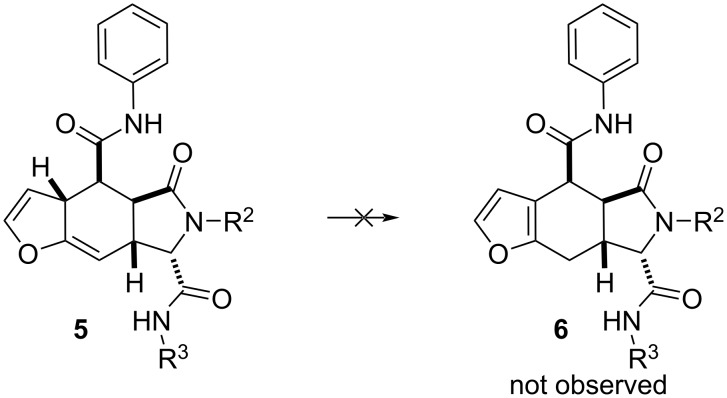
Kinetic product **5** does not transform into thermodynamic product **6**.

Noteworthy, the primary kinetic product of the cycloaddition reaction **5** is not transformed into the thermodynamic product **6** via a H-shift at the last stage. The expected aromatization with the formation of a furan ring, as happens in similar reactions, does not occur (Scheme 2).

**Scheme 3:**
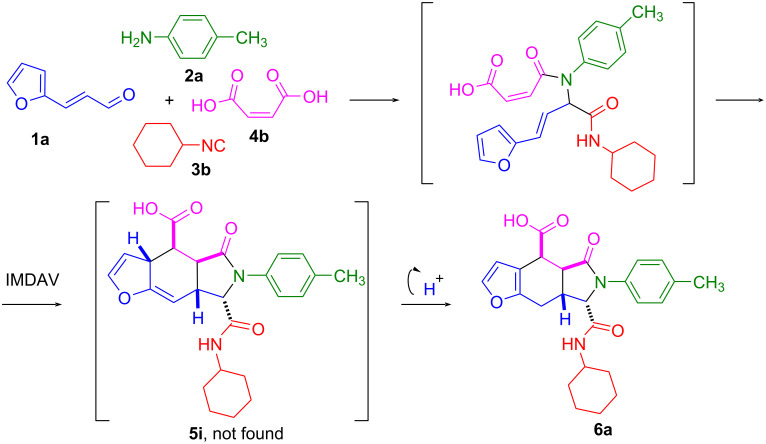
Synthesis of compounds **6a**.

However, if maleic acid was used instead of its monoanilide, then an aromatic furan product was obtained (Scheme 3). Most likely, the presence of a carboxyl group near the hydrogen in the 3-position of the furan ring contributes to its shift and isomerization to a more thermodynamically beneficial aromatic product.

**Scheme 4:**
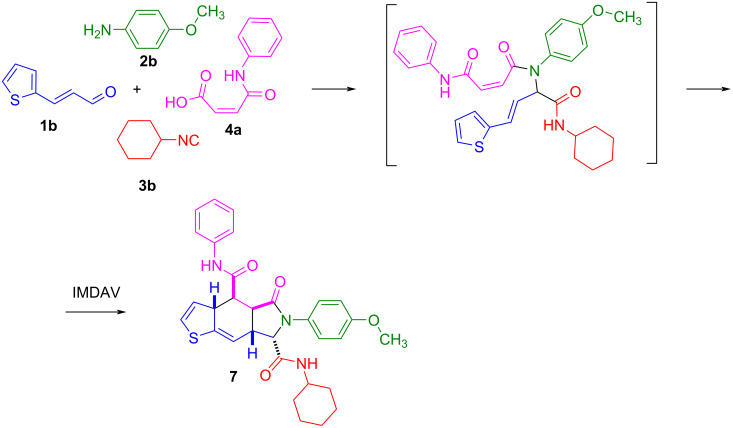
Synthesis of compound **7**.

Since we previously studied the reaction of 3-(thien-2-yl)allylamines with maleic anhydride [30], followed by a domino sequence involving successive acylation/[4 + 2] cycloaddition steps, that leads to the formation of the thieno[2,3-*f*]isoindole core, it was interesting to investigate the possibility of obtaining related compounds in the Ugi/IMDAV reaction. It was found that the reaction of (*E*)-3-(thiophen-2-yl)acrylaldehyde proceeds similarly to furylacrylaldehyde and with a high level of stereoselectivity, forms a single pair of enantiomers of 4,4a,5,6,7,7a-hexahydro-3a*H*-thieno[2,3-*f*]isoindoles in excellent yield (Scheme 4). The H-shift does not take place at the last stage, which correlates with the results obtained earlier. This shows the possibility of replacing the furan ring with a thiophene one, which significantly expands the possibilities of this approach for achieving molecular diversity of derivatives for creating libraries of compounds for bioscreening.

**Scheme 5:**
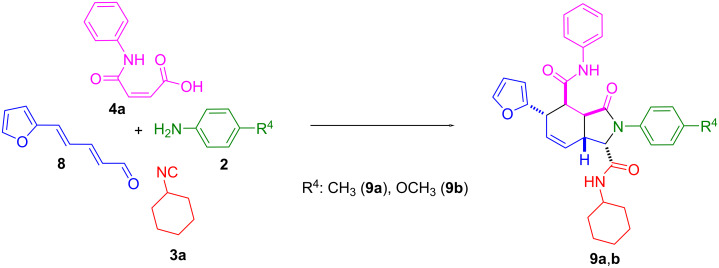
Synthesis of compounds **9**.

Finally, in such a tandem Ugi/IMDA reaction, furyl-2-pentadienal was studied. It was found, that the Ugi adduct formed from (2*E*,4*E*)-5-(furan-2-yl)penta-2,4-dienal (**8**) underwent the IMDA reaction, without involving the furan core in the cycloaddition, thus leading to the 2,3,3a,4,5,7a-hexahydro-1*H*-isoindole core **9**. In the IMDA stage, the exocyclic diene system is involved (Scheme 5). The yields of the products listed below for the Ugi/Diels–Alder tandem reactions were 73% (**9a**) and 70% (**9b**). The structures of these compounds were confirmed by NMR spectroscopy (see Supporting Information File 1).

## Conclusion

In summary, we showed the practical usage of 3-(furan-2-yl)acrylaldehyde in the four-component Ugi reaction with the prospect of further intramolecular [4 + 2] cycloaddition of the Ugi reaction product. We managed to carry out these two tandem reactions in one-pot, and, thus, proposed a new variant of the Ugi/Diels–Alder tandem reaction, which is highly variable and promising for implementation in combinatorial synthesis.

## Supporting Information

File 1 Experimental procedures, compound characterizations, and NMR spectra.

## Data Availability

All data that supports the findings of this study is available in the published article and/or the supporting information of this article.
