# Supplementary material for: New tandem Ugi/intramolecular Diels–Alder reaction based on vinylfuran and 1,3-butadienylfuran derivatives
Source: Beilstein J Org Chem. 2025 Feb 26;21:444–50. doi: 10.3762/bjoc.21.31 (PMC11878150; doi:10.3762/bjoc.21.31)
Supplement: File 1 — Experimental procedures, compound characterizations, and NMR spectra. [file Beilstein_J_Org_Chem-21-444-s001.pdf]

BEILSTEIN JOURNAL OF ORGANIC CHEMISTRY

Supporting Information

for

New tandem Ugi/intramolecular Diels–Alder reaction based on vinylfuran and 1,3-butadienylfuran derivatives

Yuriy I. Horak, Roman Z. Lytvyn, Andrii R. Vakhula, Yuriy V. Homza,
Nazariy T. Pokhodylo and Mykola D. Obushak

Beilstein J. Org. Chem. **2025,** *21,* 444–450. doi:10.3762/bjoc.21.31

Experimental procedures, compound characterizations, and NMR spectra

License and Terms: This is a supporting information file under the terms of the Creative Commons Attribution License (https://creativecommons.org/licenses/by/4.0). Please note that the reuse, redistribution and reproduction in particular requires that the author(s) and source are credited and that individual graphics may be subject to special legal provisions.
The license is subject to the *Beilstein Journal of Organic Chemistry* terms and conditions: (https://www.beilstein-journals.org/bjoc/terms)

Table of contents

General information S2

NMR data discussion S2

General procedure for the synthesis of Ugi/intramolecular Diels–Alder reaction products **5a**–**h**.....S4

Characterization data of synthesized compounds S4

^{1}H NMR, ^{13}C NMR of products **5a**–**h**, **6a**, **7**,and **9** S10

General information

Commercially available reagents and solvents were used without further purification. ^{1}H and ^{13}C NMR spectra were recorded on Varian Unity Plus 400 (400 and 101 MHz, respectively) and Bruker 170 Avance 500 (500 and 126 MHz, respectively) spectrometers in DMSO-d_6 solutions using TMS as internal reference. Mass spectral analyses were performed using an an Agilent 1100 series LC/MSD with API-ES/APCI mode (200 eV). The IR spectra were recorded on a Shimadzu IRSpirit-T spectrometer. Elemental analyses were accomplished using a PerkinElmer CHN Analyzer 2400 series. Melting points were determined on a Boetius melting point apparatus.

NMR data discussion

The analysis of spectral data shows that these compounds are formed in the form of two enantiomers. This result indicates that the Ugi and Diels–Alder reactions occur in a concerted manner and the tandem process is stereoselective. In the ^{1}H NMR spectra, proton signals are well separated, which makes it possible to assign them (Figure S1).

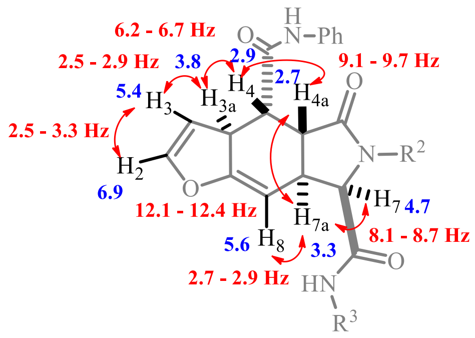

Figure S1. Vicinal spin coupling constants (^{3}J) between protons of the furoisoindole fragment, average values (red) and chemical shifts (σ values are given in ppm) (blue).

Figure S1 visualizes the vicinal spin–spin interactions of protons of the furoisondole core and the average values of chemical shifts for them (blue). It is convenient to start the analysis of the spectra with the weakest field doublet of aliphatic protons at 4.7 ppm, which belongs to the 7-H atom. This assignment is obvious because it is surrounded by acceptors: the nitrogen atom of the pyrrolidone ring and the carboxamide group. The value of the ^{3}J-coupling constant for this doublet is in the range of 8.1–8.7 Hz, which indicates the cis arrangement of the 7-H and 7a-H protons. In turn, the 7a-H proton resonates at ≈ 3.3 ppm and is usually a doublet of doublets of doublets with three ^{3}J-coupling constants within 12.1–12.4, 8.1–8.7, and 2.7–2.9 Hz, or a multiplet (if there is no fine spectrum structure). Judging by the values of the ^{3}J-coupling constant, the 7a-H proton interacts not only with 7-H, but also with the proton placed in the trans position of 4a-H, and with the 8-H proton, the dihedral angle between the H–C–C–H bond planes is close to 90°, as a result of which the J-coupling constant

is small. The signals of 8-H and 4a-H protons are at ≈ 5.6 and 2.7 ppm, respectively, which is generally characteristic of vinylidene and aliphatic protons in such an environment. In turn, the 4a-H proton interacts with the 4-H proton placed in the cis-position, with a frequency of 9.1–9.7 Hz. At the same time, the spin–spin interaction constants of 4-H and 3a-H protons, despite the trans location, are within 6.2–6.7 Hz. At first glance, this result does not seem obvious, but it can be understood if a space model of such compounds is considered (Figure S2) and the dihedral angles are measured (Table S1) between the corresponding planes of H–C–C–H bonds. Continuing the analysis of spin interactions, we see that the 3a-H, 3-H, and 2-H protons interact with each other with rather small values of the ^3J (2.5–3.3 Hz), which is generally characteristic of 2,3-dihydrofuran systems. It should be noted separately that in the ^{1}H NMR spectra of the synthesized compounds, we sometimes observe long-range spin–spin interactions, which is probably due to the inflexibility of the furoindole skeleton.

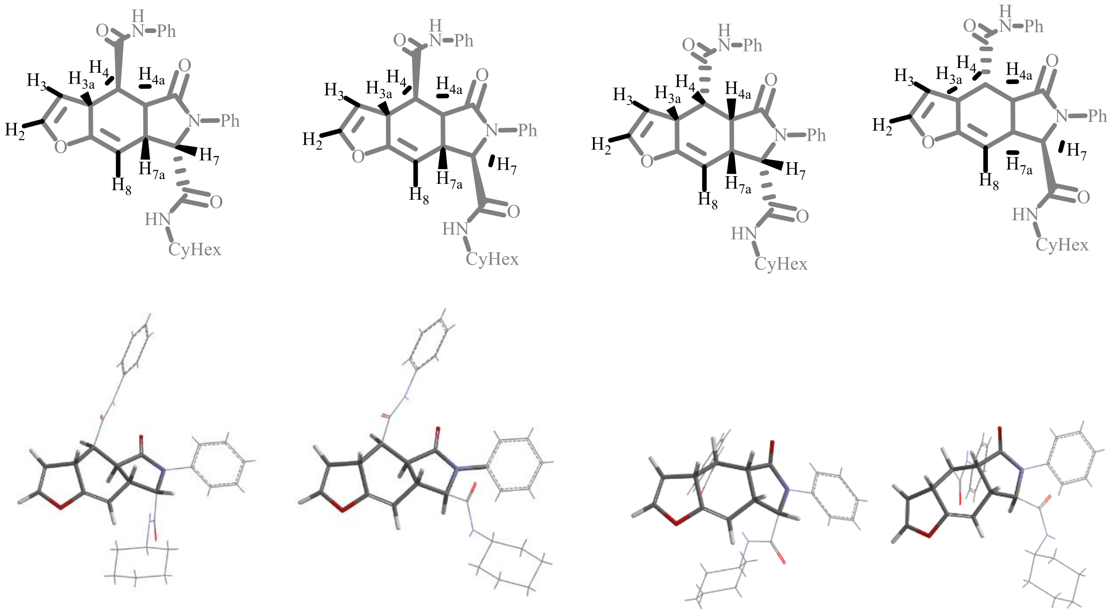

“7S-exo” **“7R-exo”** **“7S-endo”** **“7R-endo”**

Figure S2. Models and structures of possible diastereomers (one enantiomer depicted for clarity), geometry optimized at the DFT/B3LYP/6.31G* level.

Table S1. Selected angles for models of different diastereomers

Dihedral angle, °	**“7S-exo”**	**“7R-exo”**	**“7S-endo”**	**“7R-endo”**
**H_7-H_{7a}**	35.30	160.39	2.96	150.80
**H_{7a}-H_{4a}**	168.96	170.85	2.48	21.75
**H_{4a}-H_4**	45.02	45.30	32.51	45.29
**H_4-H_{3a}**	128.58	128.24	48.23	52.12

General procedure for the synthesis of Ugi/intramolecular Diels–Alder reaction products 5a–h

To a solution of 3.3 mmol of the corresponding aldehyde **1**, **8** in 25 mL of ethanol was added 3.3 mmol of amine **2**, 3.3 mmol (0.57 g) of maleic acid monoanilide (**4**), and 3.4 mmol of isonitrile **3**, and the mixture was heated for 5–6 h. The resulting precipitate was filtered off, washed with ethanol and recrystallized from an ethanol/DMF/water mixture.

Compounds **6**, **7**, and **9** were obtained by a similar procedure.

Characterization data of synthesized compounds

*(3aRS,4RS,4aSR,7SR,7aRS)-N^7-Butyl-6-(4-methoxyphenyl)-5-oxo-N^4-phenyl-4,4a,5,6,7,7a-hexahydro-3aH-furo[2,3-f]isoindole-4,7-dicarboxamide (**5a**)*

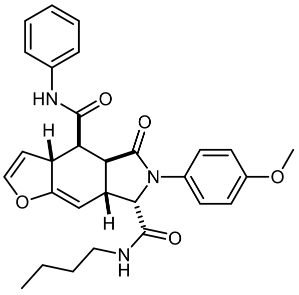

White solid, 1.34 g, 81% yield, m.p.: 255-256°C. IR (ATR, cm^{-1}): *ν* 3293.1, 1697.2, 1650.1, 1600.2, 1514.6, 1443.3, 1379.1, 1259.3, 1212.2, 1182.3, 1143.8, 1071.0, 1025.4, 897.0, 822.9, 753.0, 693.1, 674.6, 594.7, 567.6, 539.1, 523.42. ^{1}H NMR (500 MHz, DMSO) δ 10.27 (s, 1H), 8.48 (t, *J* = 5.6 Hz, 1H), 7.62 (d, *J* = 8.4 Hz, 2H), 7.36 (d, *J* = 9.2 Hz, 2H), 7.30 (t, *J* = 7.6 Hz, 2H), 7.05 (t, *J* = 7.4 Hz, 1H), 6.91-6.88 (m, 3H), 5.58 (td, *J* = 2.7, 1.2 Hz, 1H), 5.36 (td, *J* = 4.0, 1.0 Hz, 1H), 4.75 (d, *J* = 8.1 Hz, 1H), 3.83 (ddq, *J* = 6.2, 4.9, 2.4 Hz, 1H), 3.74-3.70 (m, 4H), 3.17 (dq, *J* = 12.8, 6.7 Hz, 1H), 3.07 (dq, *J* = 12.8, 6.9 Hz, 1H), 2.92 (dd, *J* = 9.2, 6.8 Hz, 1H), 2.72 (dd, *J* = 12.4, 9.2 Hz, 1H), 1.43-1.39 (m, 2H), 1.31-1.26 (m, 2H), 0.87 (t, *J* = 7.3 Hz, 3H). ^{13}C NMR (126 MHz, DMSO) *δ* 172.19, 171.77, 168.03, 158.04, 156.03, 145.83, 139.05, 132.25, 128.60, 123.30, 122.01, 119.45, 113.83, 106.48, 95.75, 62.08, 55.21, 47.28, 45.56, 41.11, 38.27, 38.12, 31.07, 19.51, 13.55. Found, %: C 69.25; H 6.12; N 8.56. $C_{29}H_{31}N_3O_5$. Calculated, %: C 69.44; H 6.23; N 8.38.

*(3aRS,4RS,4aSR,7SR,7aRS)-N^7-Butyl-6-(4-ethoxyphenyl)-5-oxo-N^4-phenyl-4,4a,5,6,7,7a-hexahydro-3aH-furo[2,3-f]isoindole-4,7-dicarboxamide (**5b**)*

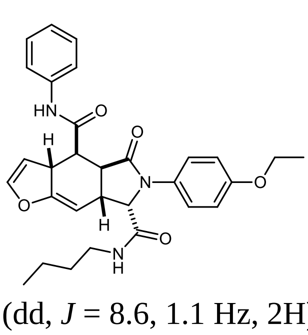

White solid, 1.28 g, 76% yield, m.p.: 267-268°C. IR (ATR, cm^{-1}): *ν* 3290.2, 2930.8, 1697.1, 1677.2, 1648.7, 1600.2, 1534.6, 1513.2, 1443.3, 1391.9, 1243.6, 1210.8, 1186.6, 1138.1, 1115.3, 1071.0, 1046.8, 1022.5, 1001.2, 922.7, 897.0, 828.6, 755.8, 711.6, 694.5, 574.7, 539.1, 510.6, 480.6. ^{1}H NMR (500 MHz, DMSO) *δ* 10.27 (s, 1H), 8.47 (t, *J* = 5.6 Hz, 1H), 7.62 (dd, *J* = 8.6, 1.1 Hz, 2H), 7.34 (d, *J* = 9.2 Hz, 2H), 7.32-7.28 (m, 2H), 7.05 (t, *J* = 7.4 Hz, 1H), 6.91-6.85 (m, 3H), 5.58 (td, *J* = 2.7, 1.2 Hz, 1H), 5.36 (td, *J* = 3.9, 1.0 Hz, 1H), 4.75 (d, *J* = 8.1 Hz, 1H), 3.98 (q, *J* = 7.0 Hz, 2H), 3.83 (ddt, *J* = 6.6, 4.0, 2.5 Hz, 1H), 3.31-3.27 (m, 1H),3.17 (dq, *J* = 12.8,

6.7 Hz, 1H), 3.07 (dq, *J* = 12.8, 6.9 Hz, 1H), 2.92 (dd, *J* = 9.2, 6.8 Hz, 1H), 2.72 (dd, *J* = 12.4, 9.2 Hz, 1H), 1.41 (pd, *J* = 7.1, 2.2 Hz, 2H), 1.31-1.23 (m, 5H), 0.87 (t, *J* = 7.3 Hz, 3H). ^{13}C NMR (126 MHz, DMSO) δ 172.19, 171.75, 168.04, 158.04, 155.28, 145.83, 139.06, 132.14, 128.60, 123.30, 121.97, 119.45, 114.33, 106.48, 95.75, 63.13, 62.07, 47.29, 45.56, 41.11, 38.26, 38.13, 31.07, 19.51, 14.59, 13.55. Found, %: C 69.67; H 6.21; N 8.34. $C_{30}H_{33}N_3O_5$. Calculated, %: C, 69.88; H, 6.45; N, 8.15.

*(3aRS,4RS,4aSR,7SR,7aRS)-N^7-Butyl-6-(4-butylphenyl)-5-oxo-N^4-phenyl-4,4a,5,6,7,7a-hexahydro-3aH-furo[2,3-f]isoindole-4,7-dicarboxamide (**5c**)*

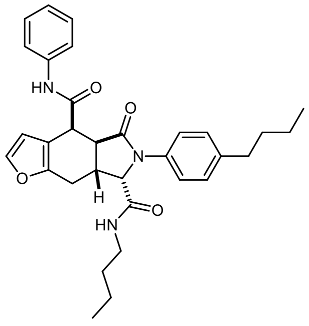

White solid, 1.42 g, 82% yield, m.p.: 204-205°C. IR (ATR, cm^{-1}): ν 3298.8, 2928.0, 2862.4, 1708.6, 1680.1, 1655.8, 1600.2, 1543.2, 1513.2, 1500.4, 1443.3, 1377.7, 1332.1, 1270.7, 1253.6, 1230.8, 1212.3, 1185.2, 1155.2, 1105.3, 1086.8, 1034.0, 897.1, 840.0, 783.0, 753.0, 738.8, 691.7, 640.4, 554.8, 522.0, 509.1. ^{1}H NMR (500 MHz, DMSO) δ 7.55 (d, *J* = 7.6 Hz, 2H), 7.52 (d, *J* = 1.5 Hz, 1H), 7.40 (d, *J* = 8.5 Hz, 2H), 7.28 (t, *J* = 7.9 Hz, 2H), 7.15 (d, *J* = 8.5 Hz, 2H), 7.04 (t, *J* = 7.4 Hz, 1H), 6.54 (d, *J* = 1.7 Hz, 1H), 4.76 (d, *J* = 8.1 Hz, 1H), 3.90 (d, *J* = 5.0 Hz, 1H), 3.78-3.69 (m, 1H), 3.19-3.13 (m, 1H), 3.11-3.04 (m, 1H), 3.02 (dd, *J* = 13.2, 5.6 Hz, 1H), 2.90 (dd, *J* = 15.3, 4.9 Hz, 1H), 2.33-2.26 (m, 1H), 1.50 (p, *J* = 7.5 Hz, 3H), 1.41 (dq, *J* = 13.4, 6.8 Hz, 2H), 1.31-1.21 (m, 5H), 0.88-0.83 (m, 6H). ^{13}C NMR (126 MHz, DMSO) δ 173.26, 170.02, 168.47, 150.65, 142.61, 139.08, 138.78, 137.04, 129.01, 128.81, 123.73, 120.06, 119.50, 116.77, 110.43, 63.04, 45.80, 38.47, 37.81, 35.27, 34.36, 33.35, 31.15, 25.06, 21.86, 19.73, 14.00, 13.80. Found, %: C 72.60; H 7.24; N 7.79. $C_{32}H_{37}N_3O_4$. Calculated, %: C 72.84; H 7.07; N 7.96.

*(3aRS,4RS,4aSR,7SR,7aRS)-N^7-Cyclohexyl-6-(4-methoxyphenyl)-5-oxo-N^4-phenyl-4,4a,5,6,7,7a-hexahydro-3aH-furo[2,3-f]isoindole-4,7-dicarboxamide (**5d**)*

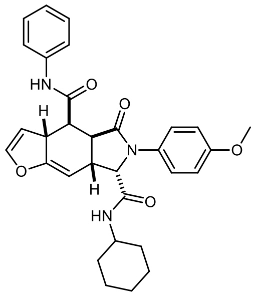

White solid, 1.36 g, 79% yield, m.p.: 270-271°C. IR (ATR, cm^{-1}): ν 3284.6, 2936.6, 2853.8, 1694.3, 1650.1, 1598.8, 1536.0, 1514.6, 1444.7, 1255.1, 1209.4, 1185.2, 1139.5, 1071.1, 1054.0, 1026.9, 1004.0, 969.8, 945.6, 892.8, 825.8, 774.4, 755.9, 723.1, 708.8, 691.7, 660.3, 594.7, 567.6, 537.7, 524.8, 504.9, 486.3, 456.4, 429.3. ^{1}H NMR (500 MHz, DMSO) δ 10.29 (s, 1H)*, 8.46 (d, *J* = 7.8 Hz, 1H)*, 7.62-7.58 (m, 2H), 7.34-7.28 (m, 4H), 7.05 (t, *J* = 7.4 Hz, 1H), 6.89 (d, *J* = 9.2 Hz, 2H), 6.87 (t, *J* = 2.7 Hz, 1H), 5.57 (td, *J* = 2.7, 1.2 Hz, 1H), 5.39-5.35 (m, 1H), 4.70 (d, *J* = 8.1 Hz, 1H), 3.81 (ddq, *J* = 6.3, 5.0, 2.4 Hz, 1H), 3.70 (s, 3H), 3.60-3.54 (m, 1H), 3.28 (ddt, *J* = 12.4, 8.0, 2.9 Hz, 1H), 2.91 (dd, *J* = 9.2, 6.8 Hz, 1H), 2.70 (dd, *J* = 12.4, 9.2

Hz, 1H), 1.79-1.54 (m, 5H), 1.28-1.15 (m, 5H); * These signals are low in intensity due to hydrogen-deuterium exchange. ^{13}C NMR (126 MHz, DMSO) δ 172.36, 172.07, 167.31, 158.20, 156.26, 146.01, 139.03, 132.42, 128.84, 123.62, 122,21, 119.62, 114.06, 106.64, 95.92, 62.09, 55.40, 47.92, 47.42, 45.72, 41.24, 38.38, 32.82, 32.29, 25.25, 24.55, 24.54. Found, %: C 70.42; H 6.18; N 7.71. $C_{31}H_{33}N_3O_5$. Calculated, %: C, 70.57; H, 6.30; N, 7.96.

*(3aRS,4RS,4aSR,7SR,7aRS)-6-(4-Chlorophenyl)-N^7-cyclohexyl-5-oxo-N^4-phenyl-4,4a,5,6,7,7a-hexahydro-3aH-furo[2,3-f]isoindole-4,7-dicarboxamide (**5e**)*

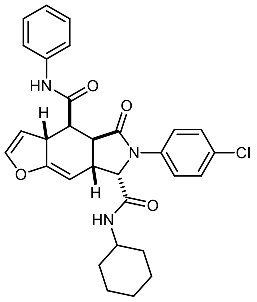

White solid, 1.29 g, 74% yield, m.p.: 325-326°C. IR (ATR, cm^{-1}): ν 3288.8, 2853.8, 1702.9, 1675.8, 1598.8, 1551.7, 1526.0, 1497.5, 1444.7, 1419.0, 1372.0, 1286.4, 1266.5, 1246.5, 1208.0, 1142.4, 1095.3, 1069.6, 1024.0, 1001.2, 966.9, 892.8, 825.8, 761.6, 750.2, 710.2, 696.0, 677.4, 657.5, 583.3, 563.3, 540.5, 513.4, 429.2. ^{1}H NMR (500 MHz, DMSO) δ 10.32 (s, 1H)*, 8.55 (d, J = 7.8 Hz, 1H)*, 7.60 (dd, J = 8.6, 1.1 Hz, 2H), 7.49 (d, J = 9.1 Hz, 2H), 7.39 (d, J = 9.1 Hz, 2H), 7.33-7.29 (m, 2H), 7.06 (t, J = 7.4 Hz, 1H), 6.87 (t, J = 2.6 Hz, 1H), 5.58 (td, J = 2.7, 1.2 Hz, 1H), 5.42-5.37 (m, 1H), 4.82 (d, J = 8.1 Hz, 1H), 3.81 (ddq, J = 6.4, 5.0, 2.5 Hz, 1H), 3.57 (tt, J = 8.2, 3.8 Hz, 1H), 3.29 (ddt, J = 11.3, 8.1, 2.9 Hz, 1H), 2.93 (dd, J = 9.2, 6.8 Hz, 1H), 2.72 (dd, J = 12.5, 9.2 Hz, 1H), 1.80-1.53 (m, 5H), 1.29-1.13 (m, 5H); * These signals are low in intensity due to hydrogen-deuterium exchange. ^{13}C NMR (126 MHz, DMSO) δ 172.61, 172.26, 166.88, 158.29, 146.04, 138.98, 138.25, 128.86, 128.16, 123.68, 121.17, 119.62, 106.62, 95.72, 61.43, 47.99, 47.55, 45.75, 41.22, 38.06, 32.77, 32.25, 25.24, 24.54, 24.51. Found, %: C 67.86; H 5.44; N 7.74. $C_{30}H_{30}ClN_3O_4$. Calculated, %: C 67.73; H 5.68; N, 7.90.

*Ethyl 4-((3aRS,4RS,4aSR,7SR,7aRS)-7-(cyclohexylcarbamoyl)-5-oxo-4-(phenylcarbamoyl)-4a,5,7,7a-tetrahydro-3aH-furo[2,3-f]isoindol-6(4H)-yl)benzoate (**5f**)*

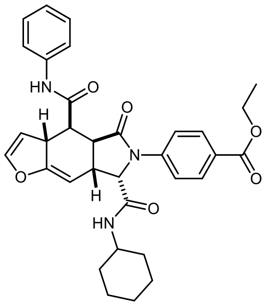

White solid, 1.51 g, 81% yield, m.p.: 303-304°C. IR (ATR, cm^{-1}): ν 3293.1, 2935.1, 2855.3, 1705.7, 1674.4, 1644.4, 1547.4, 1516.1, 1444.7, 1426.2, 1366.3, 1277.9, 1246.5, 1230.8, 1205.1, 1189.5, 1142.4, 1113.9, 1071.1, 1025.4, 1001.2, 968.4, 894.2, 854.3, 838.6, 815.8, 767.3, 755.9, 694.6, 674.6, 581.9, 563.3, 540.5, 512.01. ^{1}H NMR (500 MHz, DMSO) δ10.35 (s, 1H)*, 8.64 (d, J = 7.8 Hz, 1H)*, 7.92 (d, J = 9.0 Hz, 2H), 7.65 (d, J = 9.0 Hz, 2H), 7.61 (d, J = 7.5 Hz, 2H), 7.33-7.29 (m, 2H), 7.07 (t, J = 7.4 Hz, 1H), 6.88 (t, J = 2.6 Hz, 1H), 5.59 (td, J = 2.7, 1.2 Hz, 1H), 5.43-5.38 (m, 1H), 4.92 (d, J = 8.2 Hz, 1H), 4.27 (q, J = 7.1 Hz, 2H), 3.81 (ddq, J = 6.4, 5.0, 2.4 Hz, 1H), 3.58 (td, J = 8.4, 7.6, 4.2 Hz, 1H), 3.31 (ddt, J = 12.3, 8.0, 2.8 Hz, 1H), 2.96 (dd, J = 9.1, 6.8 Hz, 1H), 2.76 (dd, J = 12.6, 9.1 Hz, 1H), 1.79-1.53 (m, 5H), 1.31-1.15

(m, 8H); * These signals are low in intensity due to hydrogen-deuterium exchange. ^{13}C NMR (126 MHz, DMSO) δ 173.38, 172.54, 167.04, 165.72, 158.65, 146.36, 143.76, 139.27, 130.54, 129.18, 125.26, 124.00, 119.92, 118.63, 106.92, 95.97, 61.49, 61.11, 48.30, 48.01, 46.07, 41.57, 38.16, 33.03, 32.54, 25.54, 24.83, 24.79, 14.63. Found, %: C 69.71; H 6.32; N 7.63. $C_{33}H_{35}N_3O_6$. Calculated, %: C, 69.58; H, 6.19; N, 7.38.

*(3aRS,4RS,4aSR,7SR,7aRS)-6-((5-(3-Chlorophenyl)furan-2-yl)methyl)-N^7-cyclohexyl-5-oxo-N^4-phenyl-4,4a,5,6,7,7a-hexahydro-3aH-furo[2,3-f]isoindole-4,7-dicarboxamide (**5g**)*

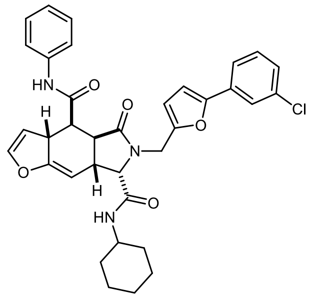

White solid, 1.60 g, 80% yield, m.p.: 292-293°C. IR (ATR, cm^{-1}): ν 3300.2, 2929.4, 2855.3, 1697.2, 1648.7, 1600.2, 1536.0, 1498.9, 1471.8, 1443.3, 1309.3, 1246.5, 1228.0, 1138.1, 1062.5, 1026.9, 891.4, 793.0, 753.0, 717.4, 691.7, 567.6, 539.1, 502.0, 449.2, 417.9. ^{1}H NMR (500 MHz, DMSO) δ 7.84 (dd, J = 7.9, 1.7 Hz, 1H), 7.61 (dd, J = 8.6, 1.1 Hz, 2H), 7.53 (dd, J = 8.0, 1.2 Hz, 1H), 7.41 (td, J = 7.6, 1.3 Hz, 1H), 7.35-7.29 (m, 5H), 7.07-7.04 (m, 2H), 6.84 (t, J = 2.7 Hz, 1H), 6.46 (d, J = 3.4 Hz, 1H), 5.54 (td, J = 2.7, 1.2 Hz, 1H), 5.29 (td, J = 3.8, 1.1 Hz, 1H), 4.62 (d, J = 15.8 Hz, 1H), 4.20 (d, J = 8.1 Hz, 1H), 4.05 (d, J = 15.7 Hz, 1H), 3.79-3.75 (m, 1H), 3.15-3.08 (m, 1H), 2.89-2.83 (m, 1H), 1.75-1.51 (m, 5H), 1.21-1.09 (m, 5H). Found, %: C 68.81; H 5.46; N 6.69. $C_{35}H_{34}ClN_3O_5$. Calculated, %: C 68.68; H 5.60; N 6.86.

*(3aRS,4RS,4aSR,7SR,7aRS)-6-((5-(4-Chlorophenyl)furan-2-yl)methyl)-N^7-cyclohexyl-5-oxo-N^4-phenyl-4,4a,5,6,7,7a-hexahydro-3aH-furo[2,3-f]isoindole-4,7-dicarboxamide (**5h**)*

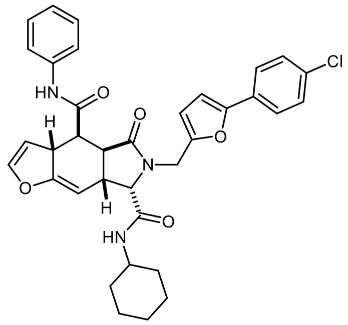

White solid, 1.44 g, 72% yield, m.p.: 268-269°C. IR (ATR, KBr, cm^{-1}): ν 3297.4, 2925.1, 2853.8, 1685.7, 1655.8, 1597.3, 1534.6, 1500.3, 1483.2, 1441.9, 1407.6, 1369.1, 1352.0, 1296.4, 1272.1, 1249.3, 1225.1, 1173.8, 1159.5, 1138.1, 1123.8, 1098.1, 1059.7, 1038.3, 1022.6, 1008.3, 998.3, 969.8, 954.1, 929.9, 902.8, 889.9, 872.8, 858.6, 832.9, 822.9, 804.4, 790.1, 767.3, 750.2, 730.2, 688.8, 670.3, 616.1, 596.1, 567.6, 556.2, 541.9, 499.1, 426.44. ^{1}H NMR (500 MHz, DMSO) δ 10.25 (s, 1H), 8.20 (d, J = 7.8 Hz, 1H), 7.70 (d, J = 8.7 Hz, 2H), 7.63 (d, J = 7.5 Hz, 2H), 7.46 (d, J = 8.7 Hz, 2H), 7.33-7.29 (m, 2H), 7.06 (t, J = 7.4 Hz, 1H), 6.94 (d, J = 3.3 Hz, 1H), 6.86 (t, J = 2.6 Hz, 1H), 6.40 (d, J = 3.3 Hz, 1H), 5.55 (td, J = 2.8, 1.2 Hz, 1H), 5.30 (td, J = 3.8, 1.0 Hz, 1H), 4.64 (d, J = 15.8 Hz, 1H), 4.20 (d, J = 8.0 Hz, 1H), 3.99 (d, J = 15.8 Hz, 1H), 3.79 (ddq, J = 6.3, 5.0, 2.4 Hz, 1H), 3.56-3.48 (m, 1H), 3.11 (ddt, J = 11.9, 8.6, 2.9 Hz, 1H), 2.86 (dd, J = 9.3, 6.8 Hz, 1H), 2.51-2.47 (1H, m)*, 1.75-1.51 (m, 5H), 1.26-1.11 (m, 5H); *Signal is overlapped with DMSO. ^{13}C NMR (126 MHz, DMSO) δ 172.39, 172.11, 166.64, 157.75, 151.76, 150.02, 145.78,139.07, 131.73, 129.09, 128.78, 128.58, 125.12, 123.31,

119.47, 110.99,107.32, 106.45, 95.96, 59.70, 48.69, 47.74, 46.22, 45.47, 40.08, 38.75, 38.51, 32.69, 32.22, 25.10, 24.41. Found, %: C 68.87; H 5.41; N 6.65. $C_{35}H_{34}ClN_3O_5$. Calculated, %: C 68.68; H 5.60; N 6.86.

*(4SR,4aSR,7SR,7aRS)-7-(Cyclohexylcarbamoyl)-5-oxo-6-(p-tolyl)-4a,5,6,7,7a,8-hexahydro-4H-furo[2,3-f]isoindole-4-carboxylic acid (**6a**)*

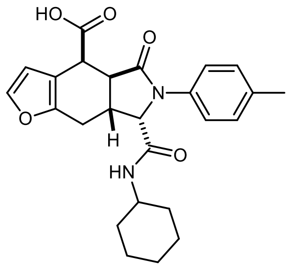

White solid, 1.07 g, 75% yield, m.p.: 267-268°C. IR (ATR, cm^{-1}): ν 3264.6, 2929.4, 2855.3, 1690.1, 1640.1, 1556.0, 1516.1, 1447.6, 1382.0, 1334.9, 1293.6, 1267.9, 1247.9, 1212.3, 1152.4, 1132.4, 1103.9, 1088.2, 942.7, 892.8, 807.2, 734.5, 668.9, 604.7, 510.6, 480.6. ^{1}H NMR (500 MHz, DMSO) δ 7.52 (d, J = 1.7 Hz, 1H), 7.40 (d, J = 8.5 Hz, 2H), 7.16 (d, J = 8.2 Hz, 2H), 6.43 (d, J = 2.0 Hz, 1H), 4.70 (d, J = 8.1 Hz, 1H), 3.77-3.74 (m, 1H), 3.60-3.53 (m, 1H)*, 3.33 (tdd, J = 13.2, 8.1, 5.1 Hz, 1H), 2.93 (dd, J = 13.2, 5.4 Hz, 1H), 2.86 (dd, J = 15.4, 5.0 Hz, 1H), 2.53-2.50 (m, 1H)**, 2.35-2.28 (m, 1H), 2.26 (s, 3H), 1.80-1.65 (m, 4H), 1.54 (d, J = 12.4 Hz, 1H), 1.31-1.14 (m, 5H); *Overlapped with water signal; **Overlapped with DMSO signal. ^{13}C NMR (126 MHz, DMSO) δ 172.99, 172.61, 167.35, 150.31, 142.52, 136.95, 133.61, 129.36, 119.82, 116.10, 110.81, 62.68, 47.86, 45.18, 36.91, 34.92, 32.62, 32.29, 25.27, 25.03, 24.49,20.53. Found, %: C 68.49; H 6.32; N 6.57. $C_{25}H_{28}N_2O_5$. Calculated, %: C 68.79; H 6.47; N 6.42.

*(3aSR,4SR,4aSR,7SR,7aSR)-N^{7}-Cyclohexyl-6-(4-methoxyphenyl)-5-oxo-N^{4}-phenyl-4,4a,5,6,7,7a-hexahydro-3aH-thieno[2,3-f]isoindole-4,7-dicarboxamide (**7**)*

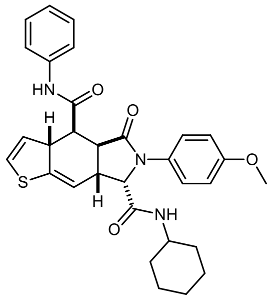

White solid, 1.41 g, 79% yield, m.p.: 307-308°C. IR (ATR, cm^{-1}): ν 3284.6, 2932.3, 2853.8, 1694.3, 1648.7, 1600.2, 1513.2, 1443.3, 1374.9, 1302.1, 1247.9, 1210.8, 1182.3, 1082.5, 1026.9, 891.4, 822.9, 805.8, 751.6, 690.3, 667.5, 583.3, 561.9, 523.4, 490.6, 419.3. ^{1}H NMR (500 MHz, DMSO) δ 10.36 (s, 1H), 8.47 (d, J = 7.9 Hz, 1H), 7.61 (d, J = 7.6 Hz, 2H), 7.34 (d, J = 9.2 Hz, 2H), 7.33-7.29 (m, 2H), 7.07 (t, J = 7.4 Hz, 1H), 6.90 (d, J = 9.2 Hz, 2H), 6.84 (d, J = 5.9 Hz, 1H), 6.29 (d, J = 6.0 Hz, 1H), 6.15 (t, J = 3.8 Hz, 1H), 4.75-4.67 (m, 2H), 3.71 (s, 3H), 3.61-3.54 (m, 1H), 3.39-3.34 (m, 1H), 3.23 (dd, J = 9.3, 6.7 Hz, 1H), 2.84 (dd, J = 13.0, 9.3 Hz, 1H), 1.83-1.53 (m, 5H), 1.32-1.14 (m, 5H). Found, %: C 68.61; H 6.32; N 7.56. $C_{31}H_{33}N_3O_4S$. Calculated, %: C 68.48; H 6.12; N 7.73.

*(1SR,3aSR,4RS,5SR,7aRS)-N¹-Cyclohexyl-5-(furan-2-yl)-3-oxo-N⁴-phenyl-2-(p-tolyl)-2,3,3a,4,5,7a-hexahydro-1H-isoindole-1,4-dicarboxamide (**9a**)*

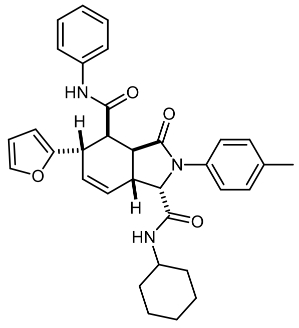

White solid, 1.29 g, 73% yield, m.p.: 283-284°C. IR (ATR, cm^{-1}): *ν* 3305.9, 2933.7, 2853.8, 1697.1, 1658.7, 1645.8, 1600.2, 1540.3, 1516.1, 1501.8, 1444.7, 1390.6, 1354.9, 1312.1, 1267.9, 1247.9, 1190.9, 1101.0, 1073.9, 1015.5, 938.4, 891.4, 867.1, 830.1, 800.1, 790.1, 751.6, 728.8, 690.3, 600.4, 556.2, 513.4, 493.4. ^{1}H NMR (500 MHz, DMSO) *δ* 9.78 (s, 1H)*, 8.39 (d, *J* = 7.8 Hz, 1H)*, 7.63 (dd, *J* = 1.8, 0.8 Hz, 1H), 7.56 (dd, *J* = 8.6, 1.1 Hz, 2H), 7.33 (d, *J* = 8.6 Hz, 2H), 7.30-7.26 (m, 2H), 7.13 (d, *J* = 8.3 Hz, 2H), 7.04 (t, *J* = 7.4 Hz, 1H), 6.42 (dd, *J* = 3.1, 1.9 Hz, 1H), 6.11 (dt, *J* = 10.0, 1.9 Hz, 1H), 6.03 (d, *J* = 3.2 Hz, 1H), 5.80 (dt, *J* = 9.9, 3.3 Hz, 1H), 4.72 (d, *J* = 7.9 Hz, 1H), 3.90-3.85 (m, 1H), 3.58-3.52 (m, 1H), 3.39 (dtd, *J* = 12.5, 4.6, 2.1 Hz, 1H), 3.34 (d, *J* = 4.3 Hz, 1H), 3.12 (dd, *J* = 13.4, 4.2 Hz, 1H), 2.23 (s, 3H), 1.78-1.65 (m, 4H), 1.54 (d, *J* = 12.4 Hz, 1H), 1.30-1.14 (m, 5H); *these signals are low in intensity due to hydrogen-deuterium exchange. ^{13}C NMR (126 MHz, DMSO) *δ* 173.29, 170.21, 166.64, 155.94, 142.56, 138.71, 136.73, 133.82, 129.39, 128.90, 128.63, 126.00, 123.72, 119.99, 119.46, 110.71, 106.85, 62.21, 47.92, 42.94, 42.29, 37.78, 36.49, 32.65, 32.20, 25.31, 24.54, 24.52, 20.51. Found, %: C 73.59; H 6.30; N 7.63. $C_{33}H_{35}N_3O_4$. Calculated, %: C 73.72; H 6.56; N 7.82.

*(1SR,3aSR,4RS,5SR,7aRS)-N¹-Cyclohexyl-5-(furan-2-yl)-2-(4-methoxyphenyl)-3-oxo-N⁴-phenyl-2,3,3a,4,5,7a-hexahydro-1H-isoindole-1,4-dicarboxamide (**9b**)*

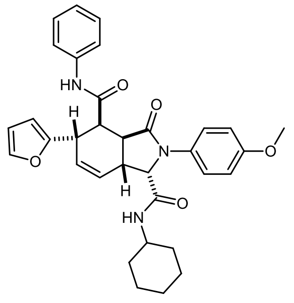

White solid, 1.28 g, 70% yield, m.p.: 282-283°C. ^{1}H NMR (500 MHz, DMSO) *δ* 9.75 (s, 1H)*, 8.35 (d, *J* = 7.8 Hz, 1H)*, 7.65-7.61 (m, 1H), 7.57-7.54 (m, 2H), 7.32 (d, *J* = 9.1 Hz, 2H), 7.30-7.26 (m, 2H), 7.04 (t, *J* = 7.4 Hz, 1H), 6.89 (d, *J* = 9.2 Hz, 2H), 6.42 (dd, *J* = 3.1, 1.9 Hz, 1H), 6.10 (d, *J* = 9.9 Hz, 1H), 6.03 (d, *J* = 3.2 Hz, 1H), 5.80 (dt, *J* = 9.9, 3.3 Hz, 1H), 4.67 (d, *J* = 7.9 Hz, 1H), 3.91-3.86 (m, 1H), 3.70 (s, 3H), 3.61-3.52 (m, 1H), 3.39 (dtd, *J* = 12.6, 4.7, 2.1 Hz, 1H), 3.34 (d, *J* = 4.3 Hz, 1H), 3.11 (dd, *J* = 13.4, 4.2 Hz, 1H), 1.78-1.65 (m, 4H), 1.54 (d, *J* = 12.2 Hz, 1H), 1.30-1.13 (m, 5H); * These signals are low in intensity due to hydrogen-deuterium exchange; ^{13}C NMR (126 MHz, DMSO) *δ* 173.15, 170.20, 166.68, 156.42, 155.95, 142.56, 138.71, 132.17, 128.91, 128.59, 126.06, 123.71, 122.36, 119.45, 114.16, 110.70, 106.86, 62.71, 55.41, 47.93, 42.98, 42.11, 37.73, 36.67, 32.67, 32.19, 25.30, 24.56, 24.53. IR (ATR, KBr, cm^{-1}): *ν* 3301.7, 2933.7, 2851.0, 1691.5, 1647.3, 1600.2, 1537.5, 1514.6, 1444.7, 1389.1, 1372.0, 1356.3, 1285.0, 1256.5, 1216.6, 1202.3, 1183.8, 1150.9, 1101.0, 1085.3, 1073.9, 1029.7, 1015.5, 938.4, 867.1, 824.3, 787.3, 750.2, 724.5, 693.1, 670.3, 596.1, 564.8, 517.7, 493.5. Found, %: C 71.32; H 6.53; N 7.42. $C_{33}H_{35}N_3O_5$. Calculated, %: C 71.59; H 6.37; N 7.59.

^{1}H NMR and ^{13}C NMR spectra of products 5a–h, 6a, 7, and 9

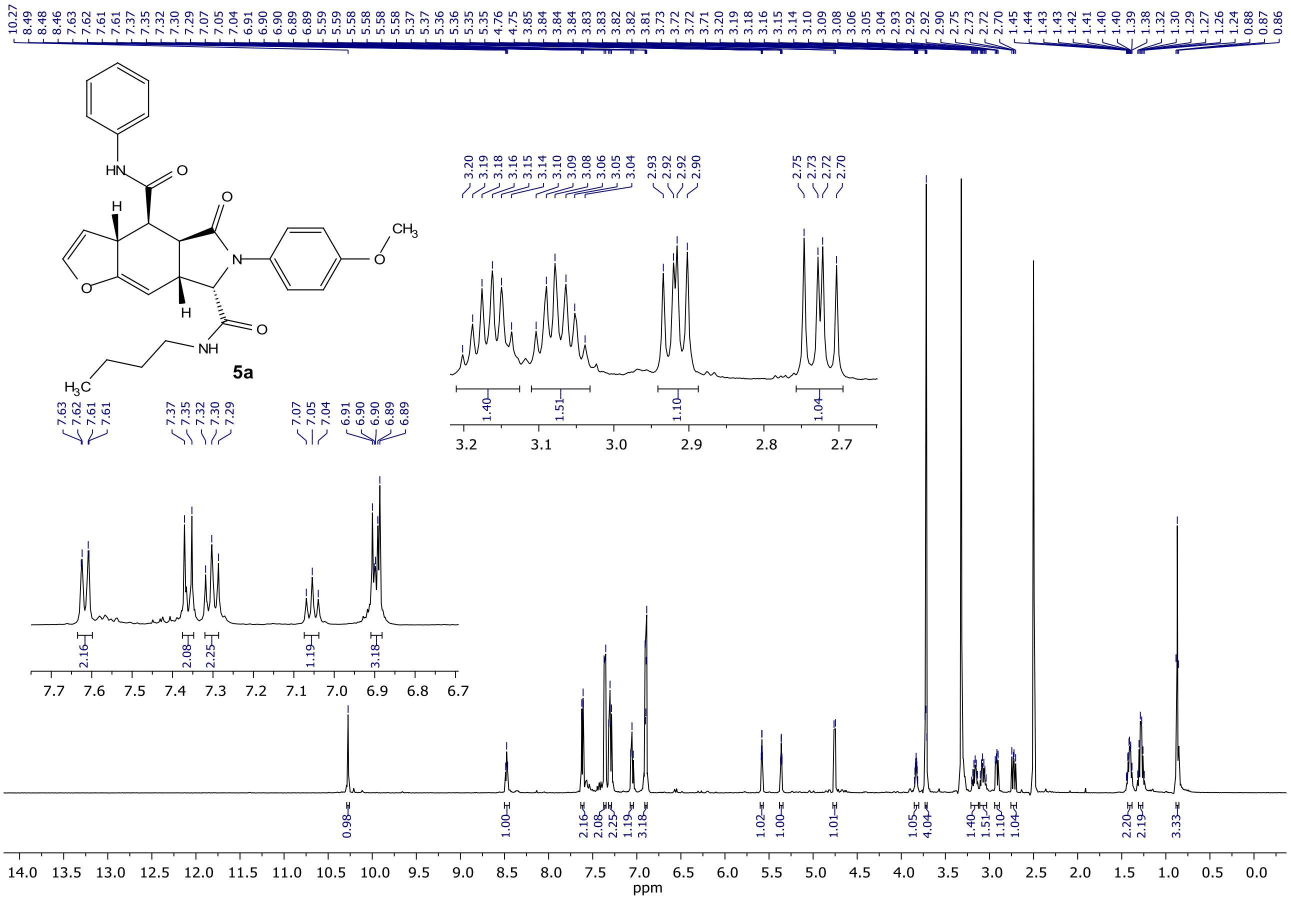

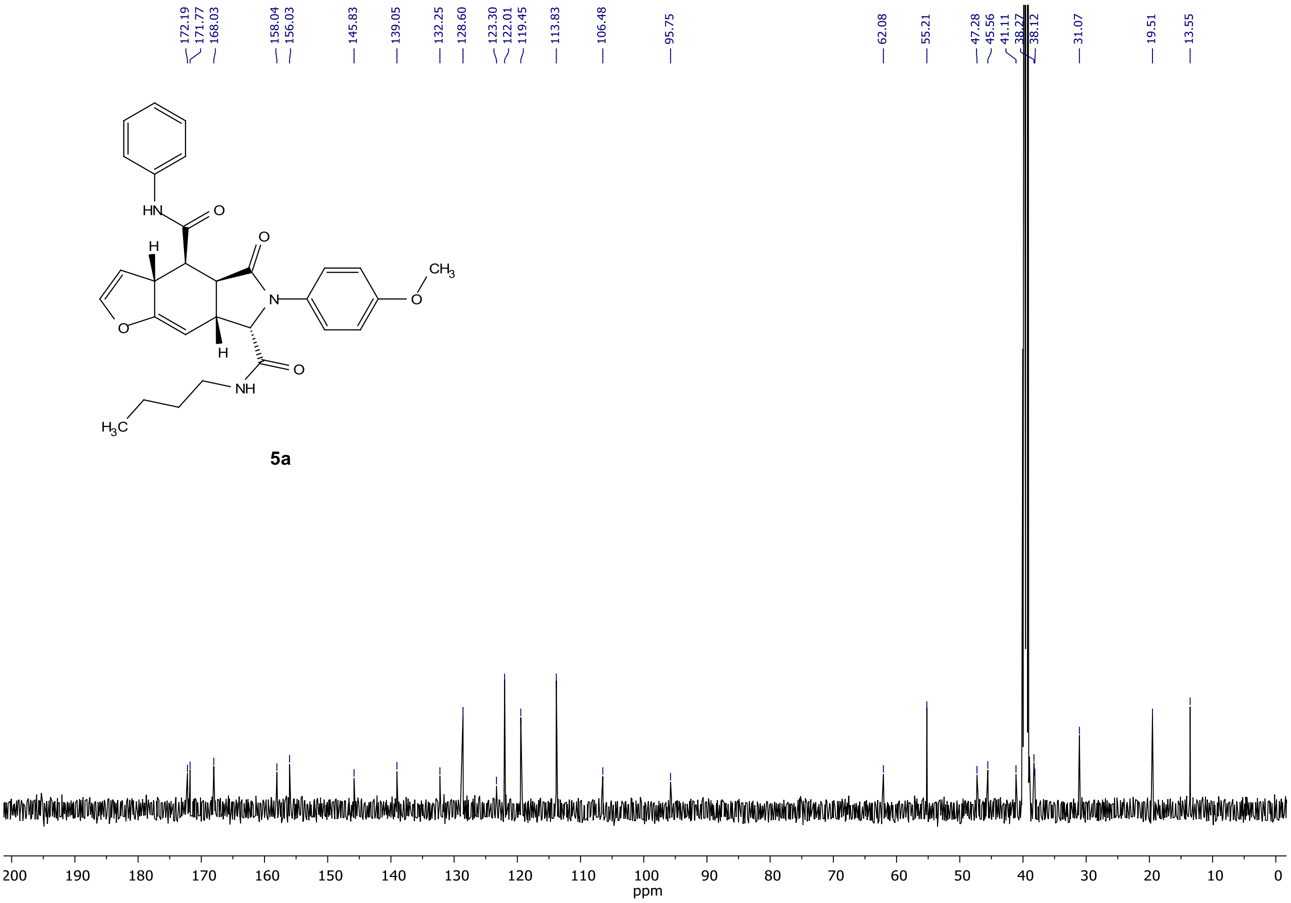

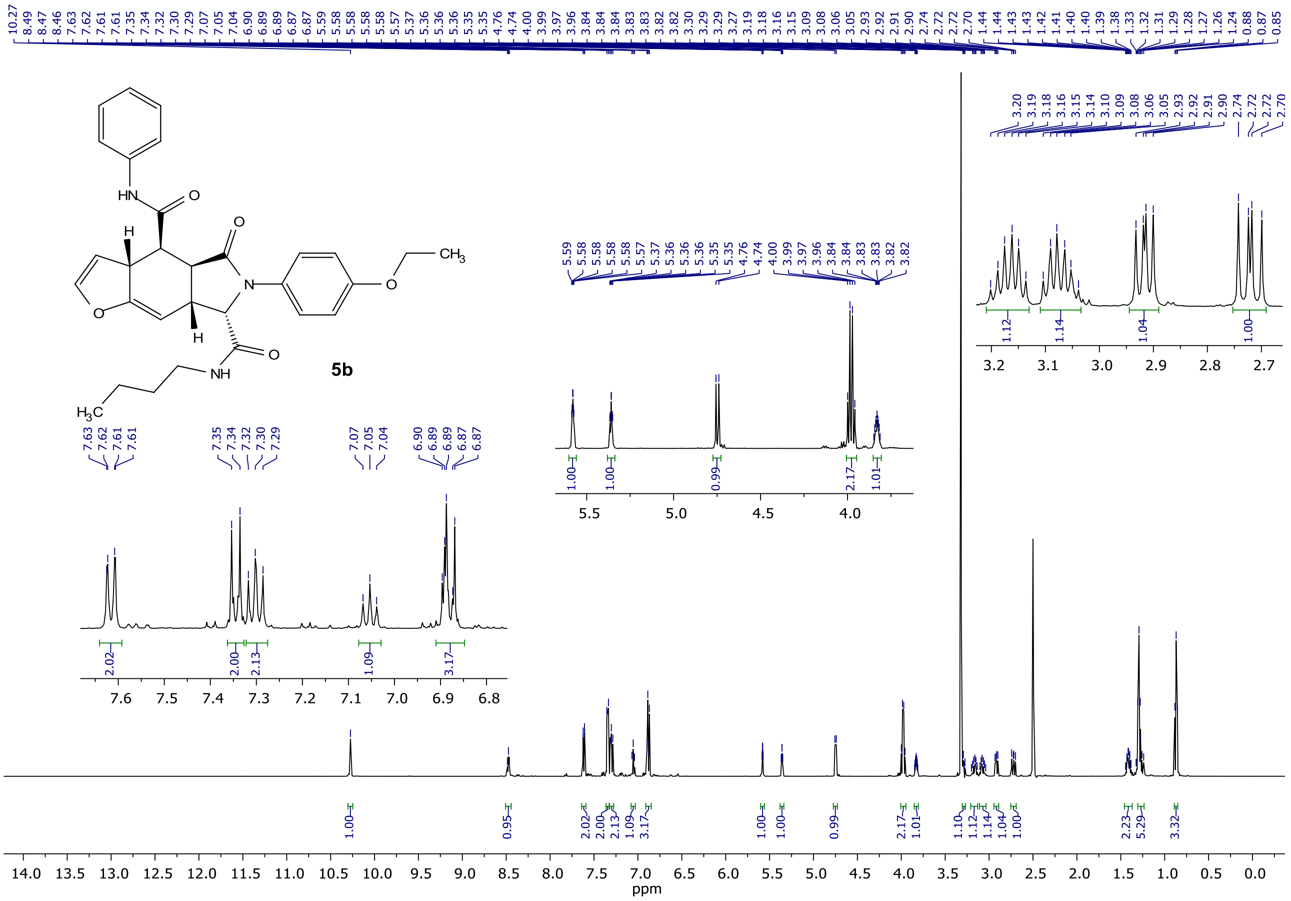
HN
O
H
O
N
CH3
O
O
H
O
NH
H3C
5b
ppm

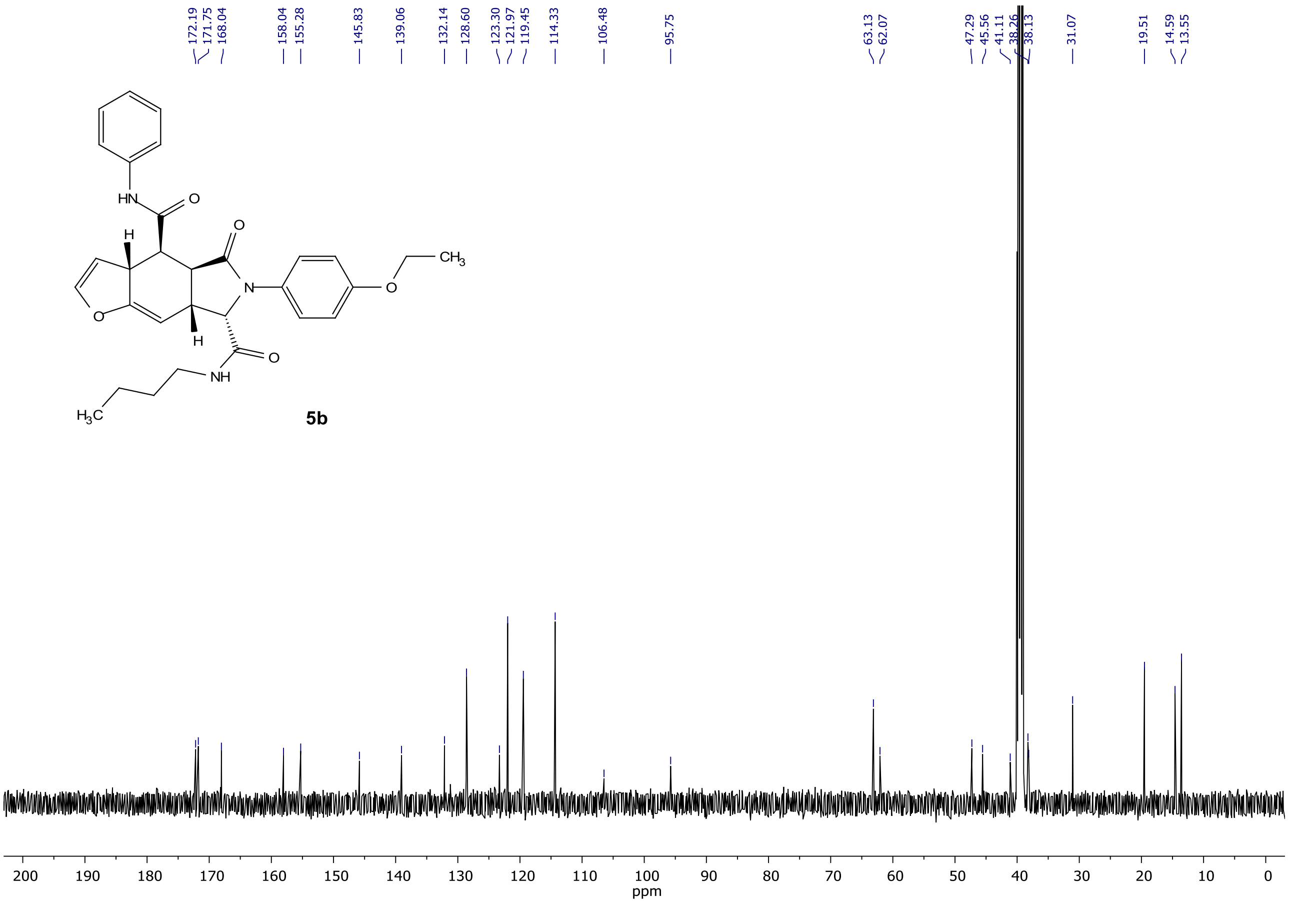
HN
O
H
O
N
CH3
O
O
H
O
NH
H3C
5b
ppm

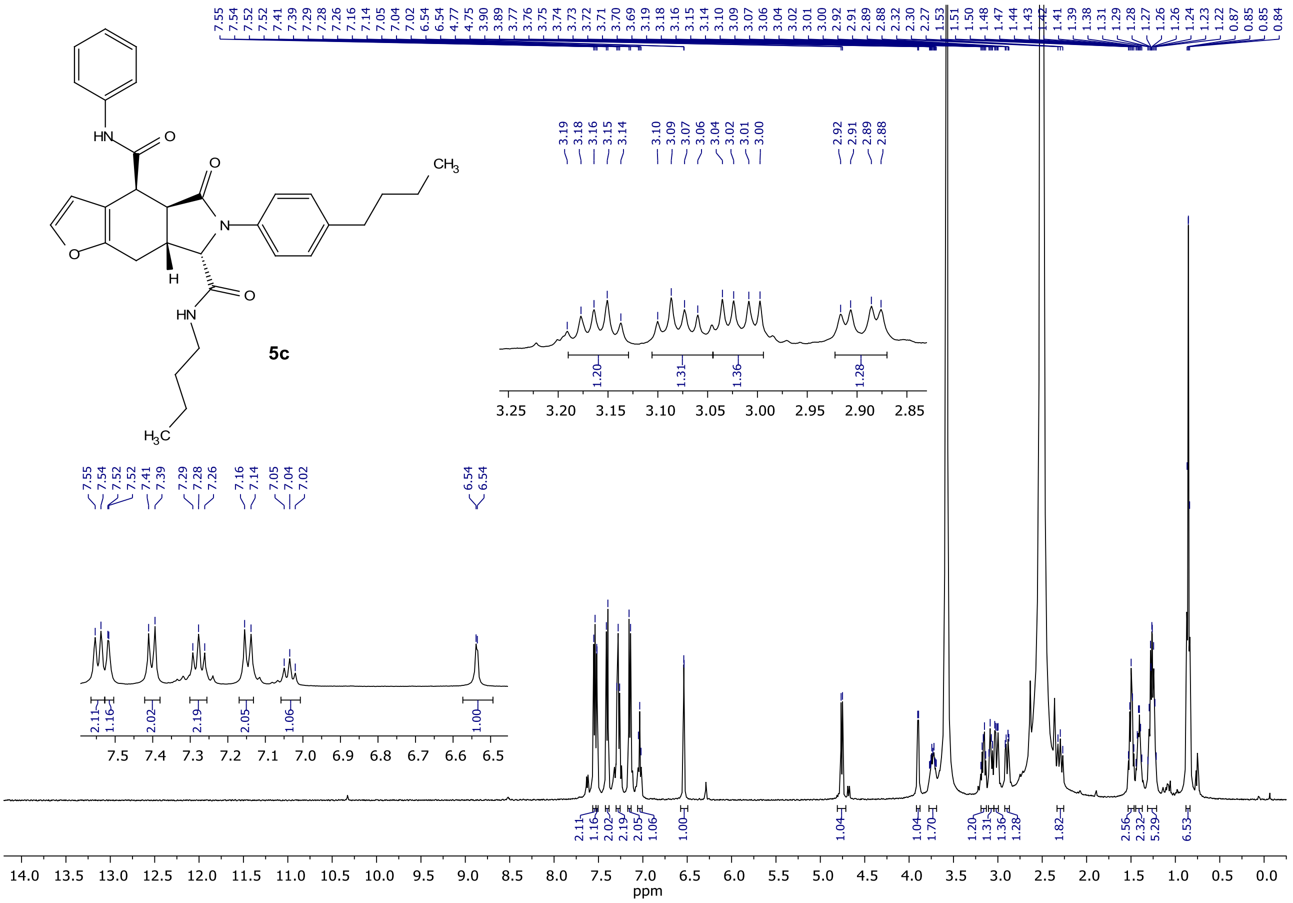
5c
ppm

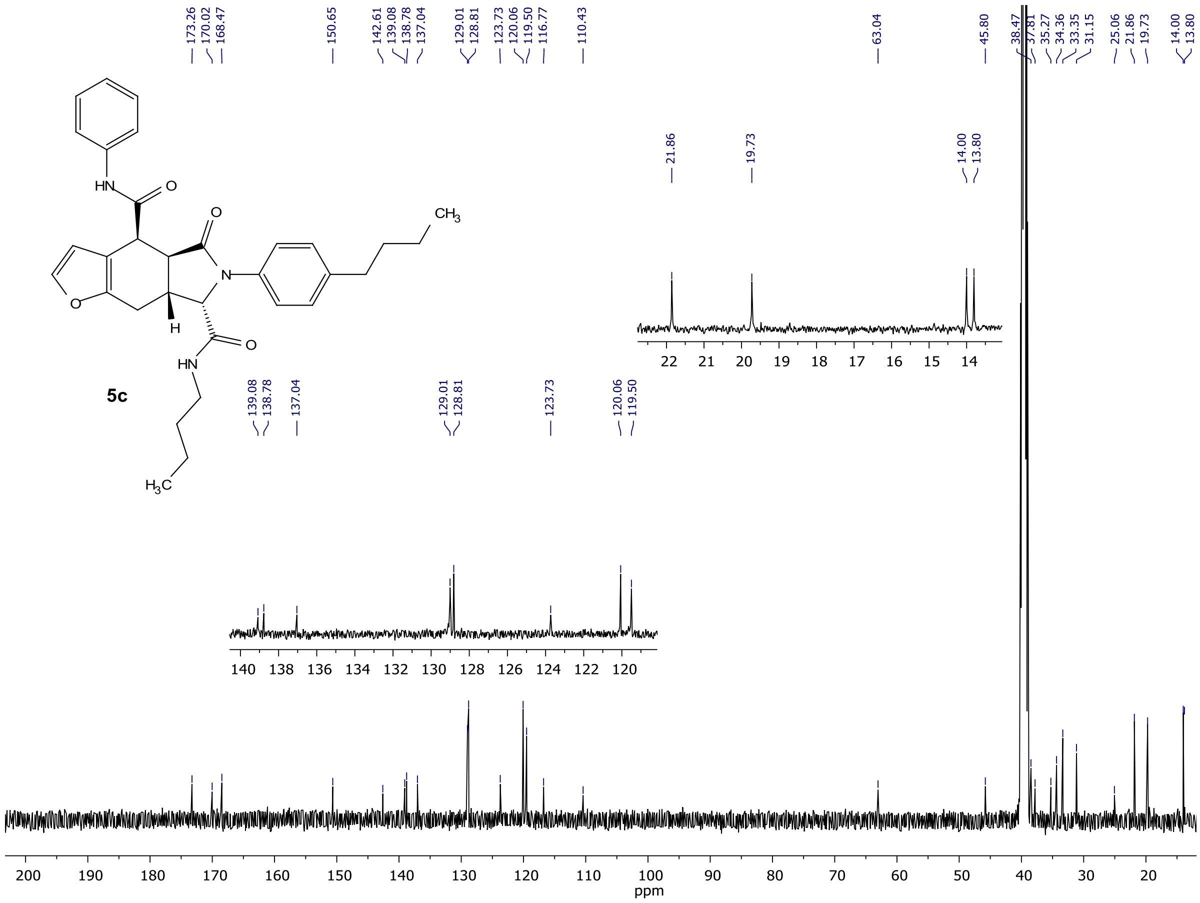
5c
ppm

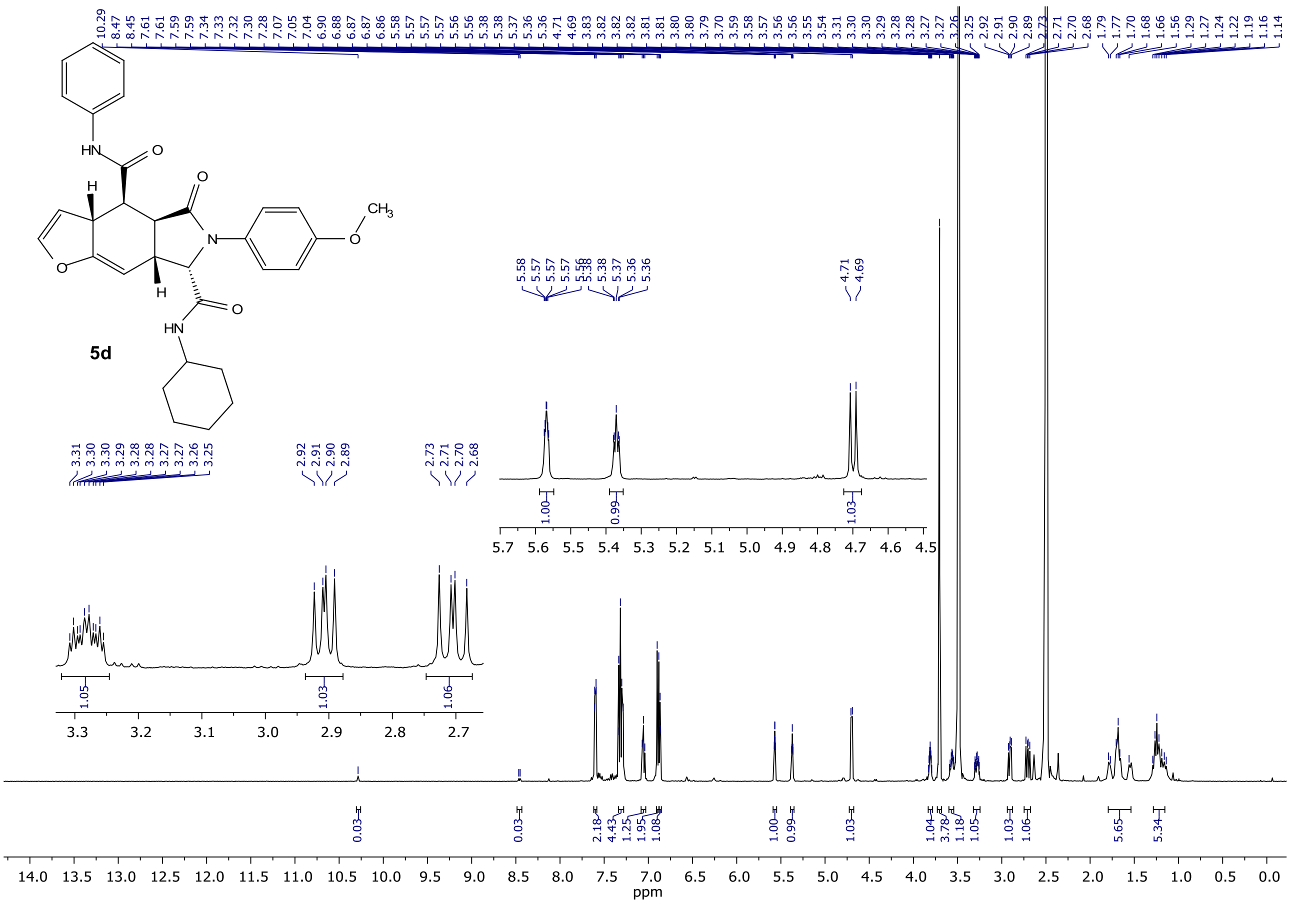
10.29 8.47 8.45 7.61 7.61 7.59 7.59 7.34 7.33 7.32 7.30 7.28 7.07 7.05 7.04 6.90 6.88 6.87 6.87 6.86 5.58 5.57 5.57 5.57 5.56 5.56 5.38 5.38 5.37 5.36 5.36 4.71 4.69 3.83 3.82 3.82 3.81 3.81 3.80 3.80 3.79 3.70 3.59 3.58 3.57 3.56 3.56 3.55 3.54 3.31 3.30 3.30 3.29 3.28 3.28 3.27 3.27 3.26 3.25 2.92 2.91 2.90 2.89 2.73 2.71 2.70 2.68 1.79 1.77 1.70 1.68 1.66 1.56 1.29 1.27 1.24 1.22 1.19 1.16 1.14
HN
O
H
O
CH3
N
O
O
H
O
HN
5d
0.03 0.03 2.18 4.43 1.25 1.95 1.08 1.00 0.99 1.03 1.04 3.78 1.18 1.05 1.03 1.06 5.65 5.34
ppm

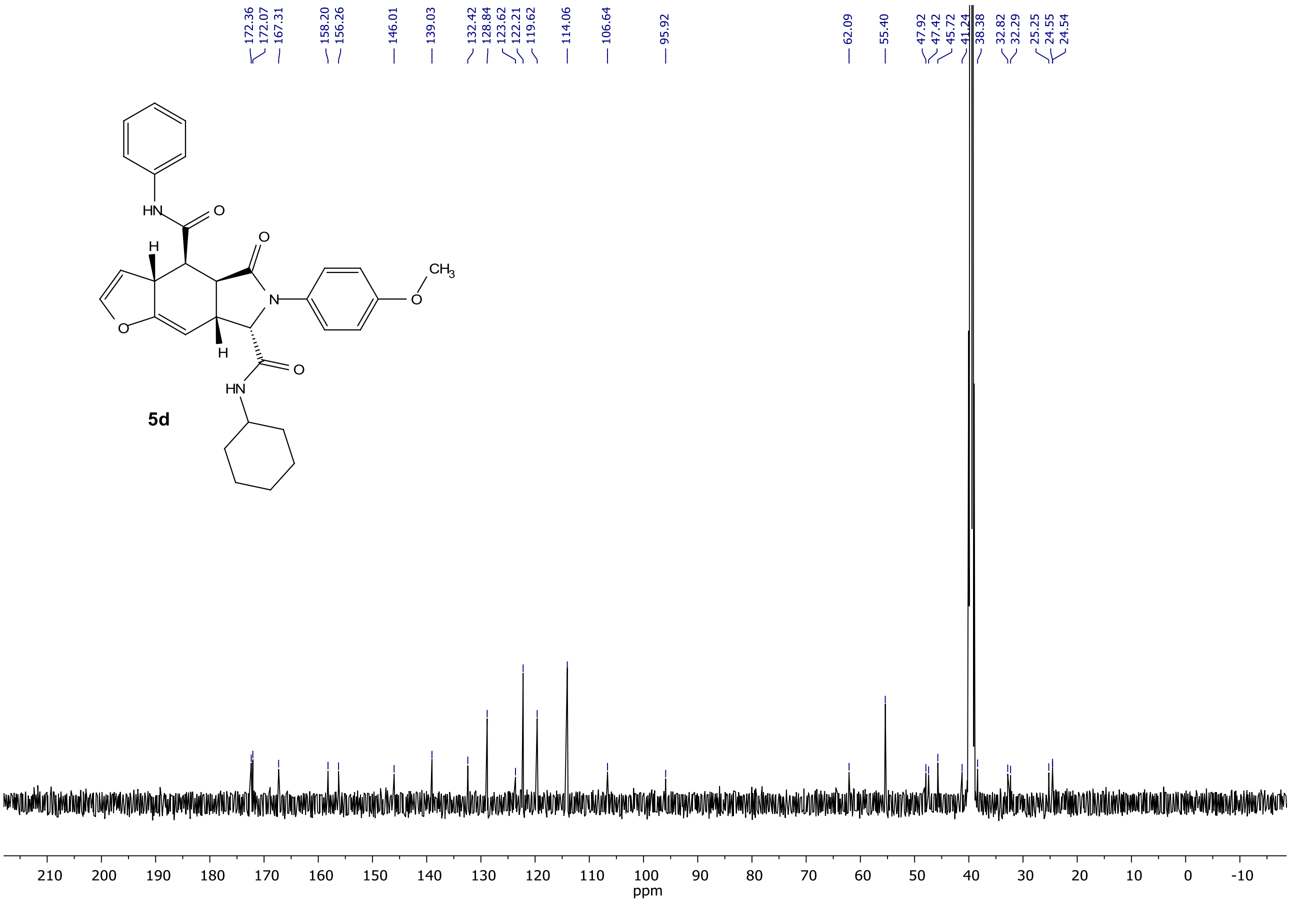
172.36 172.07 167.31 158.20 156.26 146.01 139.03 132.42 128.84 123.62 122.21 119.62 114.06 106.64 95.92 62.09 55.40 47.92 47.42 45.72 41.24 38.38 32.82 32.29 25.25 24.55 24.54
HN
O
H
O
CH3
N
O
O
H
O
HN
5d
210 200 190 180 170 160 150 140 130 120 110 100 90 80 70 60 50 40 30 20 10 0 -10
ppm

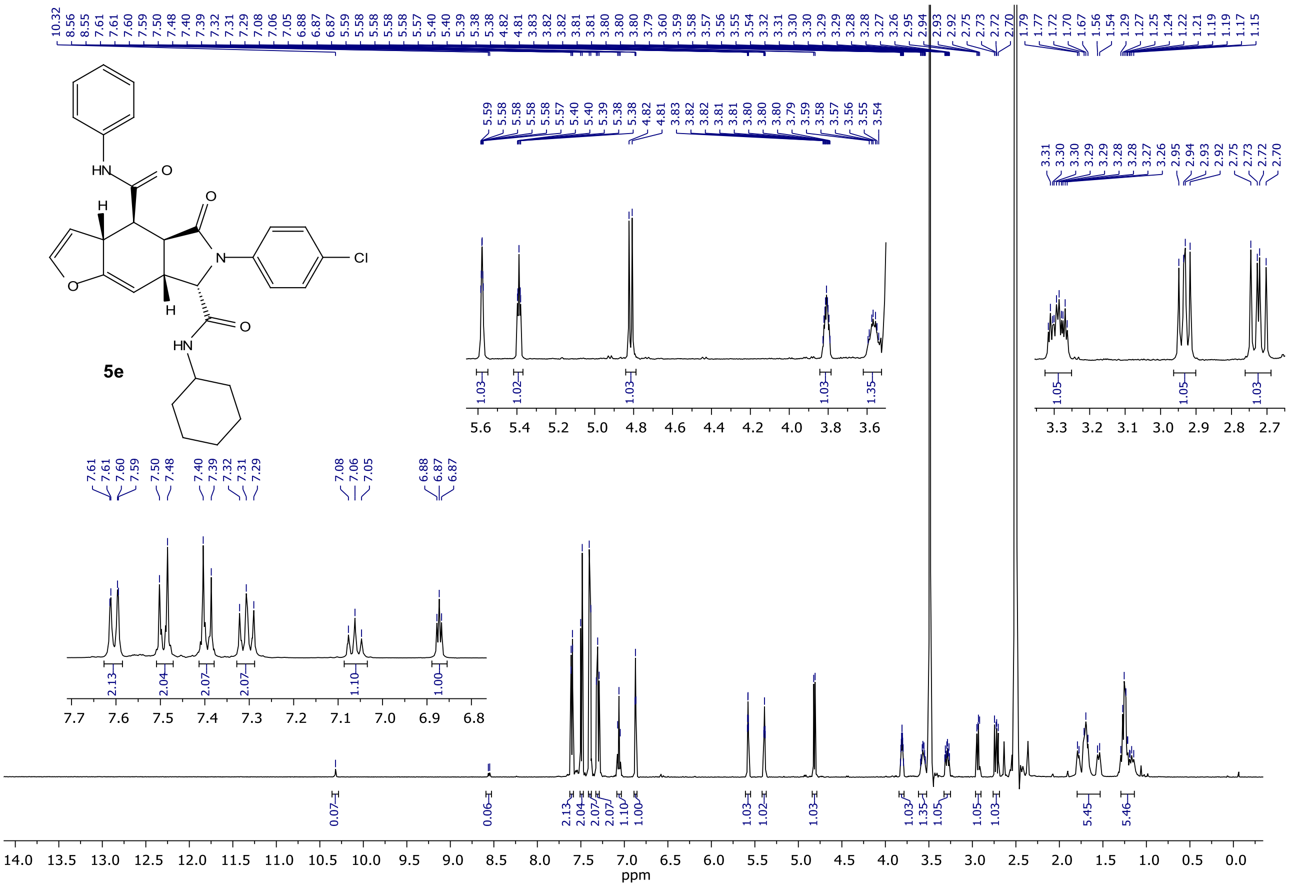
10.32 8.56 8.55 7.61 7.61 7.60 7.59 7.50 7.48 7.40 7.39 7.32 7.31 7.29 7.08 7.06 7.05 6.88 6.87 6.87 5.59 5.58 5.58 5.58 5.58 5.57 5.40 5.40 5.39 5.38 5.38 4.82 4.81 3.83 3.82 3.82 3.81 3.81 3.80 3.80 3.80 3.79 3.60 3.59 3.58 3.57 3.56 3.55 3.54 3.32 3.31 3.30 3.30 3.29 3.29 3.28 3.28 3.27 3.26 2.95 2.94 2.93 2.92 2.75 2.73 2.72 2.70 1.79 1.77 1.72 1.70 1.67 1.56 1.54 1.29 1.27 1.25 1.24 1.22 1.21 1.19 1.19 1.17 1.15
HN
O
H
O
N
Cl
O
H
O
HN
5e
0.07 0.06 2.13 2.04 2.07 2.07 1.10 1.00 1.03 1.02 1.03 1.03 1.35 1.05 1.05 1.03 5.45 5.46
14.0 13.5 13.0 12.5 12.0 11.5 11.0 10.5 10.0 9.5 9.0 8.5 8.0 7.5 7.0 6.5 6.0 5.5 5.0 4.5 4.0 3.5 3.0 2.5 2.0 1.5 1.0 0.5 0.0
ppm

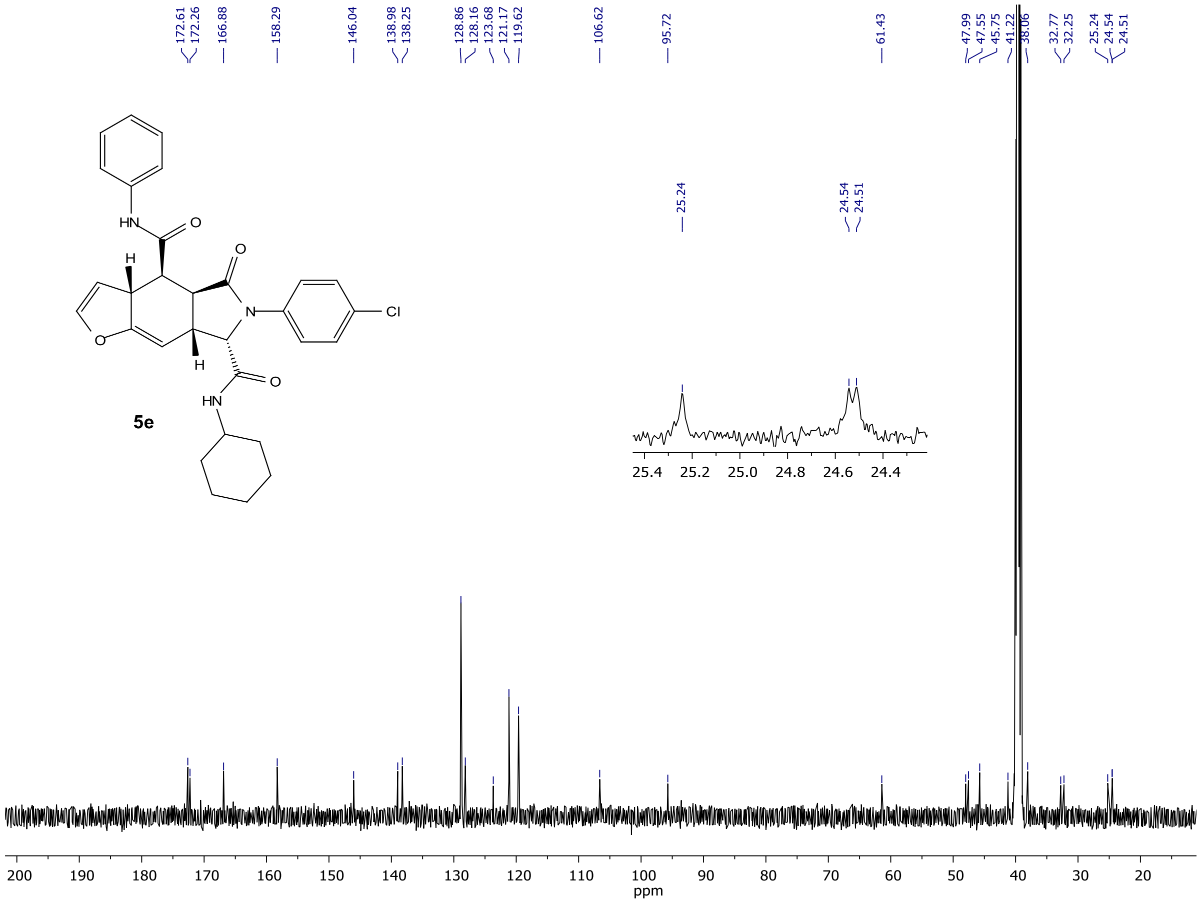
172.61 172.26 166.88 158.29 146.04 138.98 138.25 128.86 128.16 123.68 121.17 119.62 106.62 95.72 61.43 47.99 47.55 45.75 41.22 38.06 32.77 32.25 25.24 24.54 24.51
HN
O
H
O
N
Cl
O
H
O
HN
5e
25.24
24.54 24.51
25.4 25.2 25.0 24.8 24.6 24.4
200 190 180 170 160 150 140 130 120 110 100 90 80 70 60 50 40 30 20
ppm

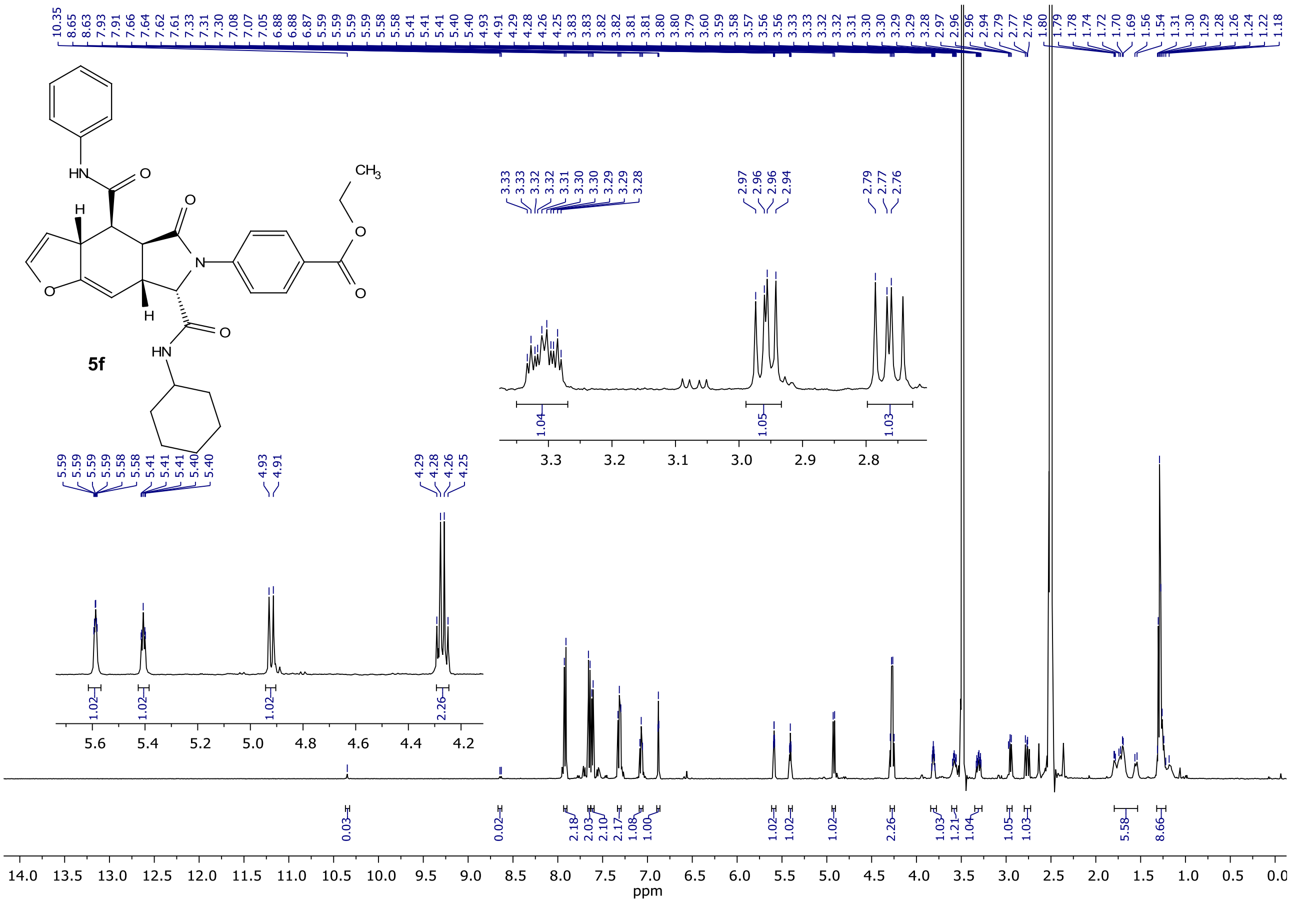
10.35 8.65 8.63 7.93 7.91 7.66 7.64 7.62 7.61 7.33 7.31 7.30 7.08 7.07 7.05 6.88 6.87 5.59 5.59 5.59 5.59 5.59 5.58 5.58 5.41 5.41 5.41 5.40 5.40 4.93 4.91 4.29 4.28 4.26 4.25 3.83 3.83 3.82 3.82 3.81 3.81 3.80 3.79 3.60 3.59 3.58 3.57 3.56 3.56 3.33 3.33 3.32 3.32 3.31 3.30 3.30 3.29 3.29 3.28 2.97 2.96 2.96 2.94 2.79 2.77 2.76 1.80 1.79 1.78 1.74 1.72 1.70 1.69 1.56 1.54 1.31 1.30 1.29 1.28 1.26 1.24 1.22 1.18
HN
O
H
O
N
O
O
O
CH3
H
HN
O
5f
0.03 0.02 2.18 2.03 2.10 2.17 1.08 1.00 1.02 1.02 1.02 2.26 1.03 1.21 1.04 1.05 1.03 5.58 8.66
ppm

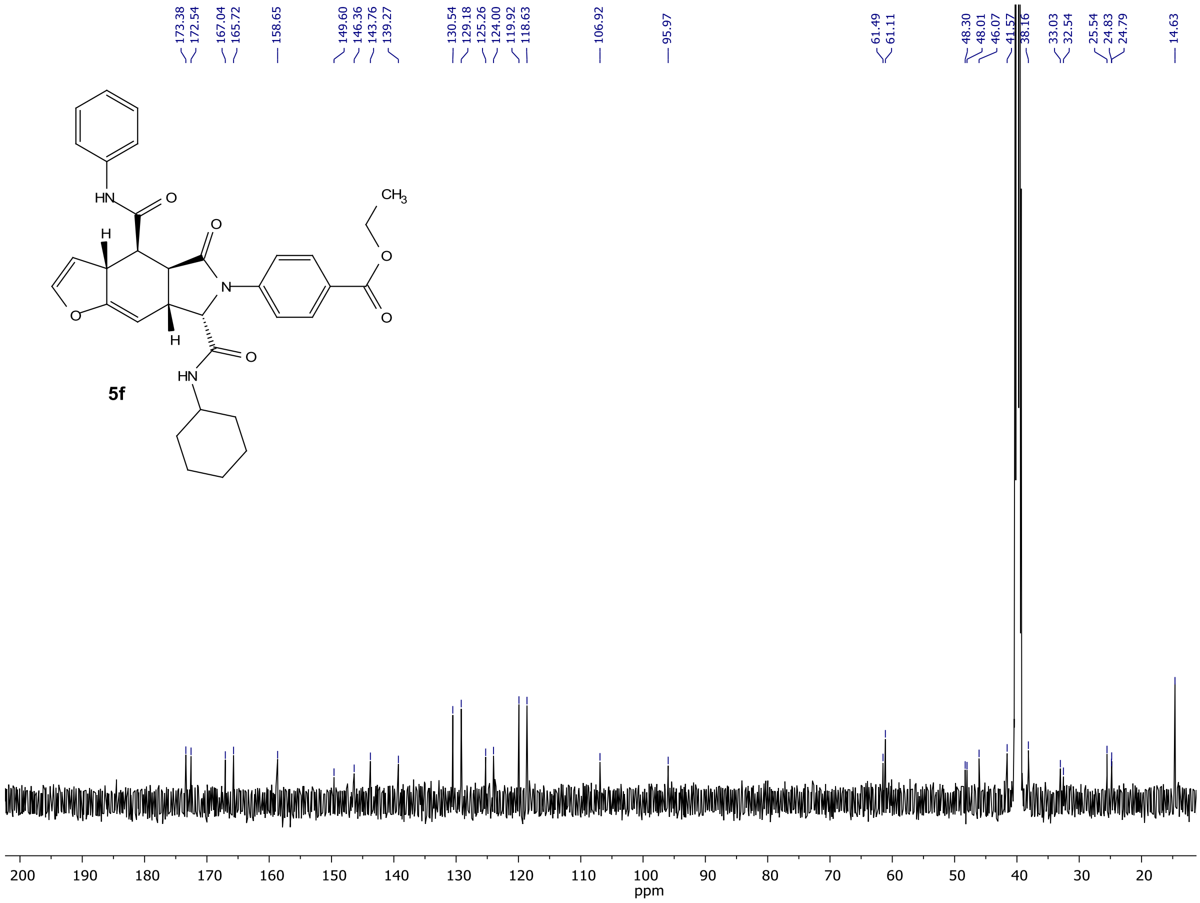
173.38 172.54 167.04 165.72 158.65 149.60 146.36 143.76 139.27 130.54 129.18 125.26 124.00 119.92 118.63 106.92 95.97 61.49 61.11 48.30 48.01 46.07 41.57 38.16 33.03 32.54 25.54 24.83 24.79 14.63
HN
O
H
O
N
O
O
O
CH3
H
HN
O
5f
ppm

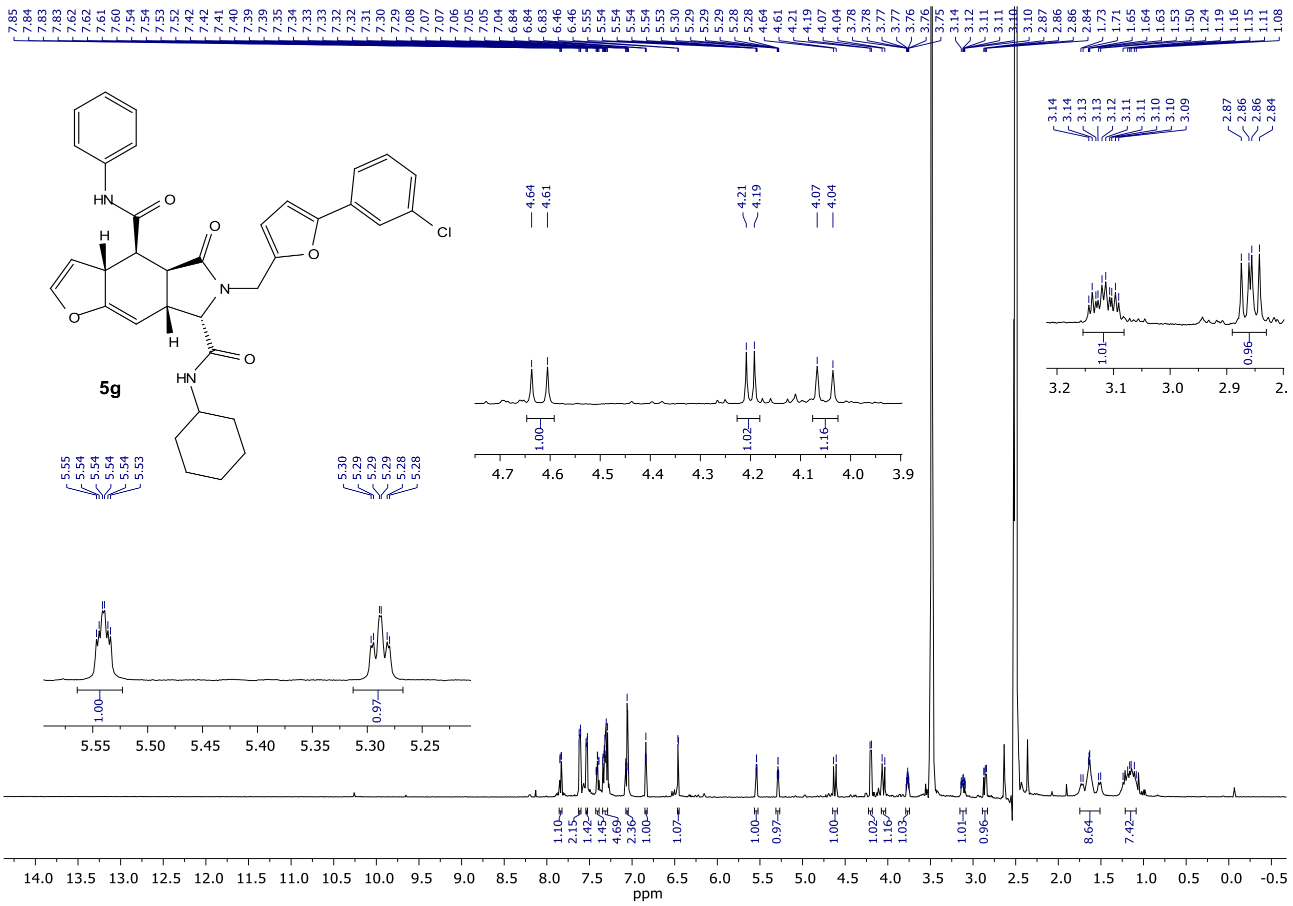

5g

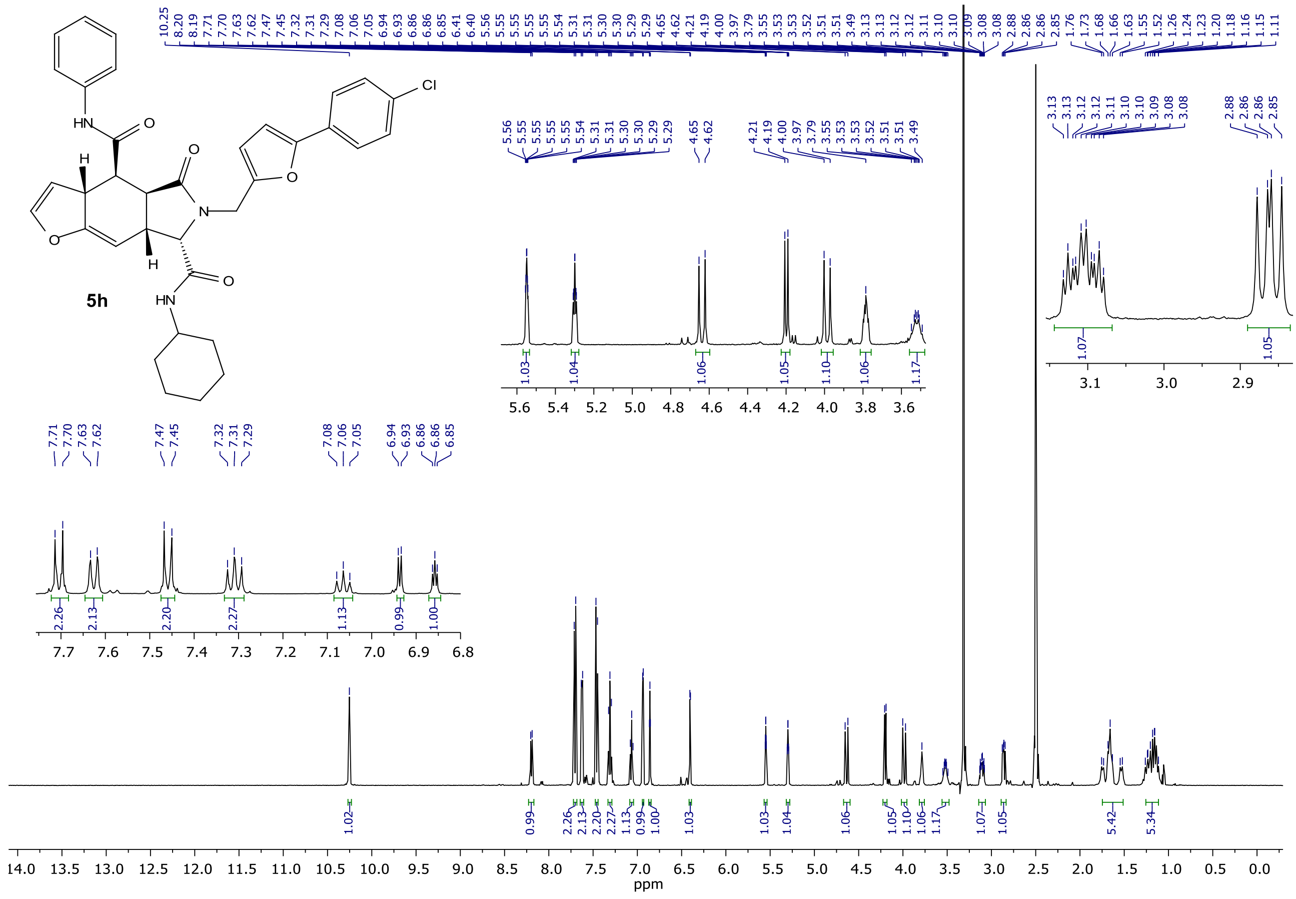

5h

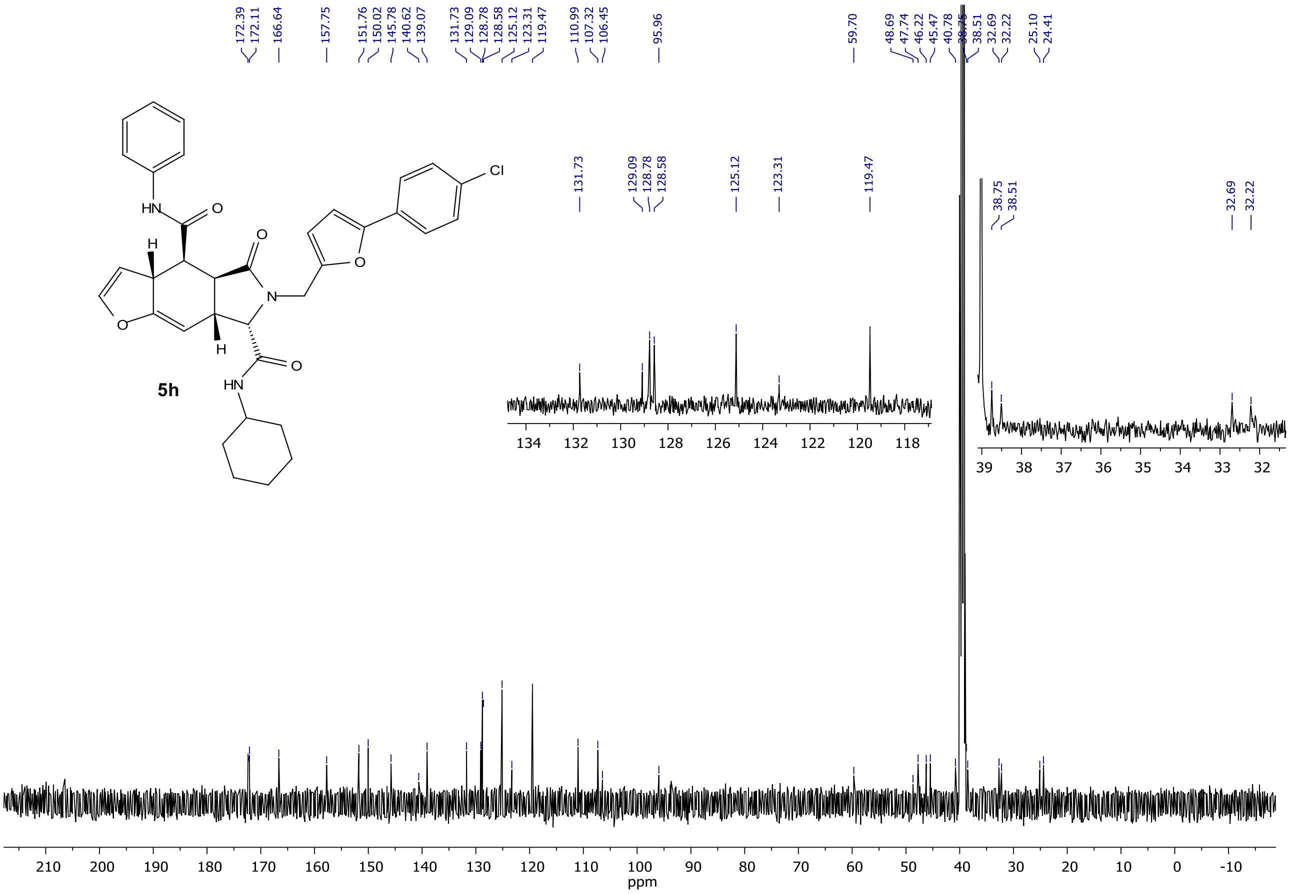
5h
Cl
172.39
172.11
166.64
157.75
151.76
150.02
145.78
140.62
139.07
131.73
129.09
128.78
128.58
125.12
123.31
119.47
110.99
107.32
106.45
95.96
59.70
48.69
47.74
46.22
45.47
40.78
38.51
32.69
32.22
25.10
24.41
38.75
ppm

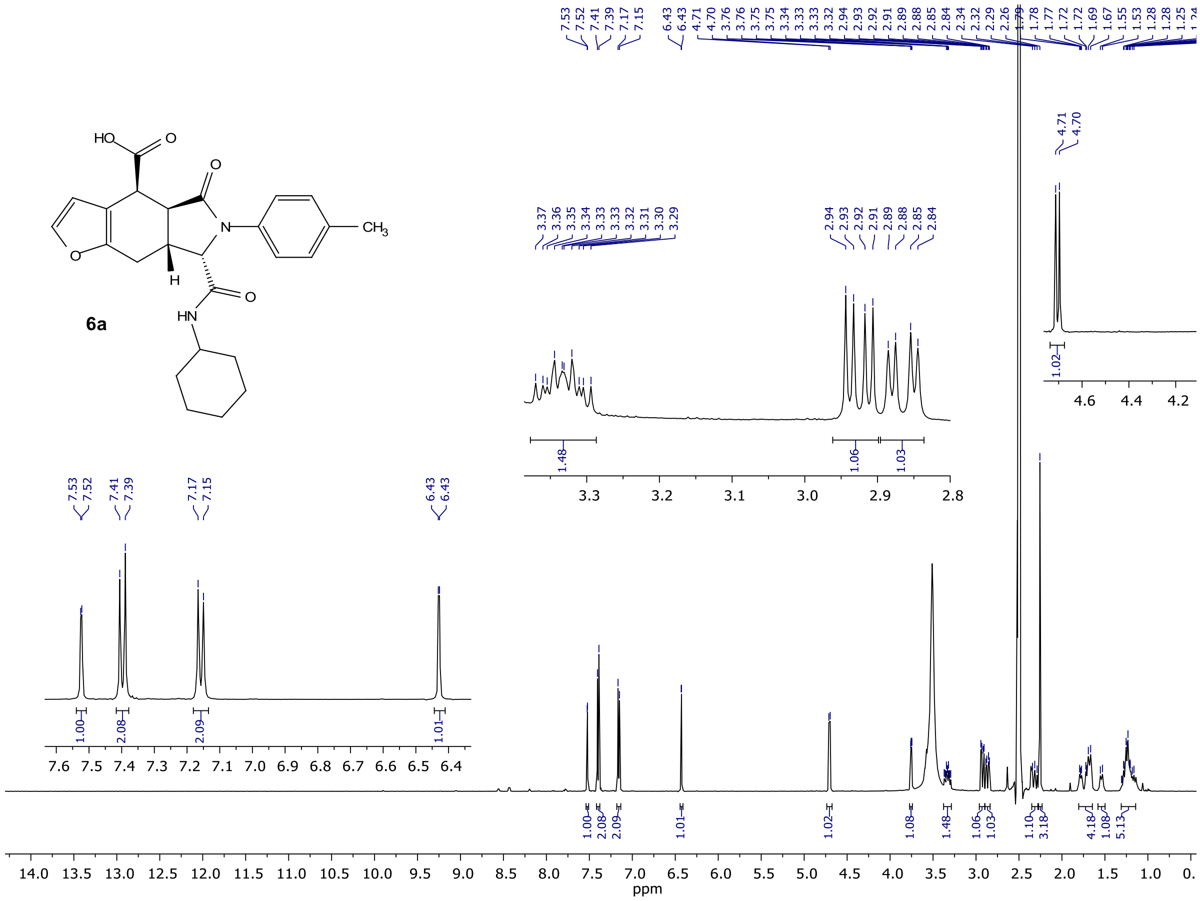
6a
HO
CH_3
7.53
7.52
7.41
7.39
7.17
7.15
6.43
6.43
4.71
4.70
3.37
3.36
3.35
3.34
3.33
3.33
3.32
3.31
3.30
3.29
2.94
2.93
2.92
2.91
2.89
2.88
2.85
2.84
ppm

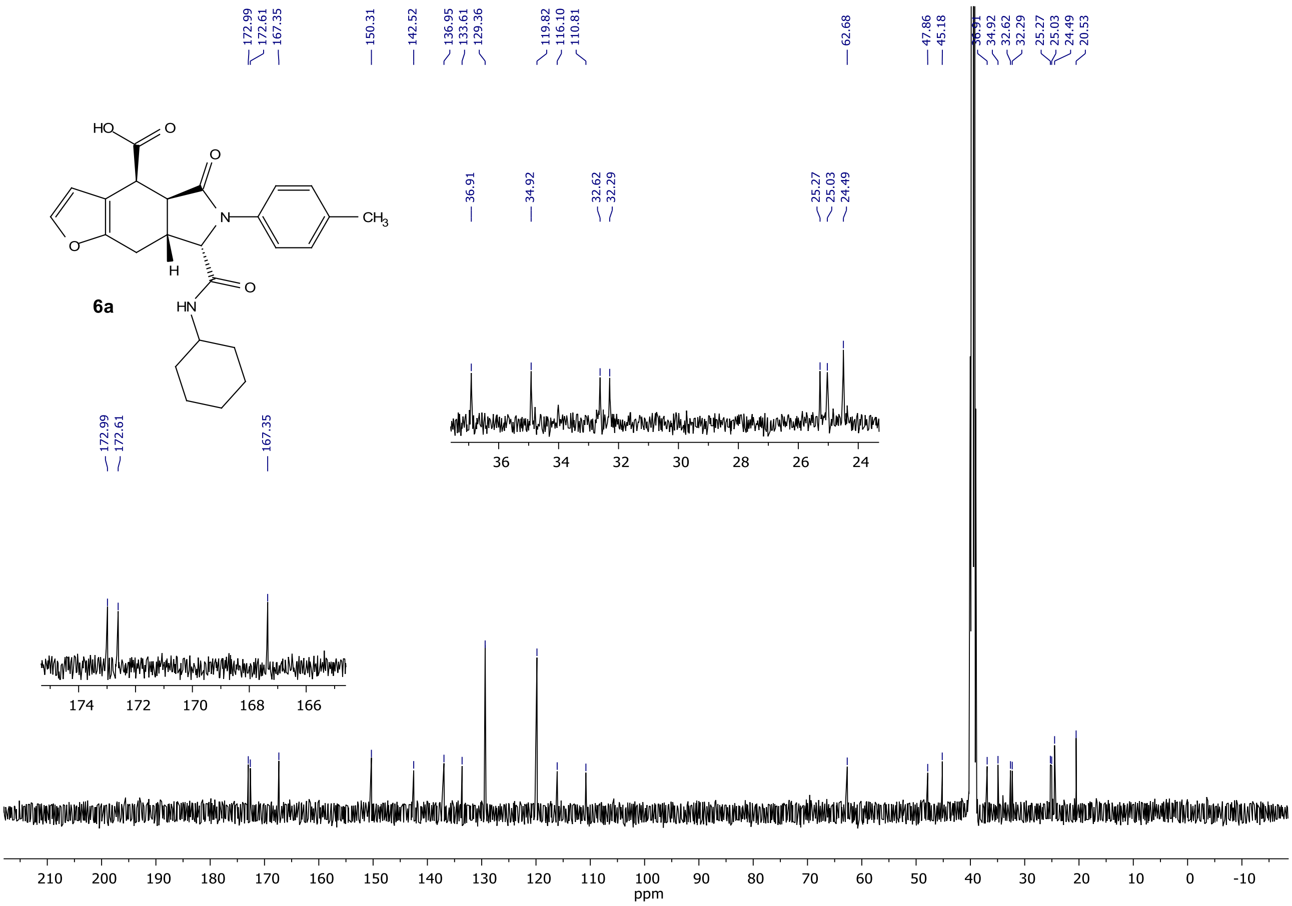
6a
172.99
172.61
167.35
150.31
142.52
136.95
133.61
129.36
119.82
116.10
110.81
62.68
47.86
45.18
36.91
34.92
32.62
32.29
25.27
25.03
24.49
20.53
ppm

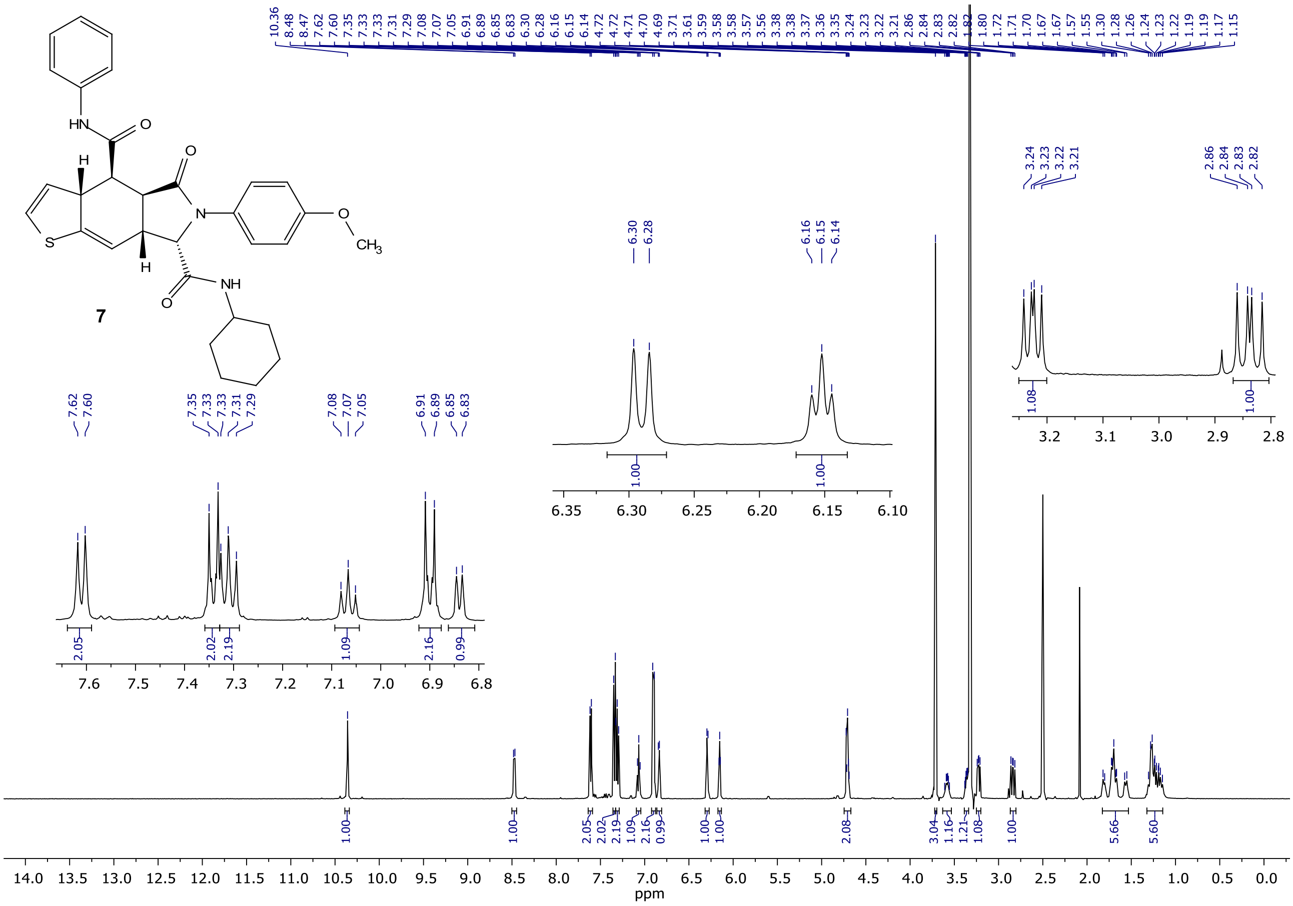
7
7.62
7.60
7.35
7.33
7.33
7.31
7.29
7.08
7.07
7.05
6.91
6.89
6.85
6.83
6.30
6.28
6.16
6.15
6.14
3.24
3.23
3.22
3.21
2.86
2.84
2.83
2.82
ppm

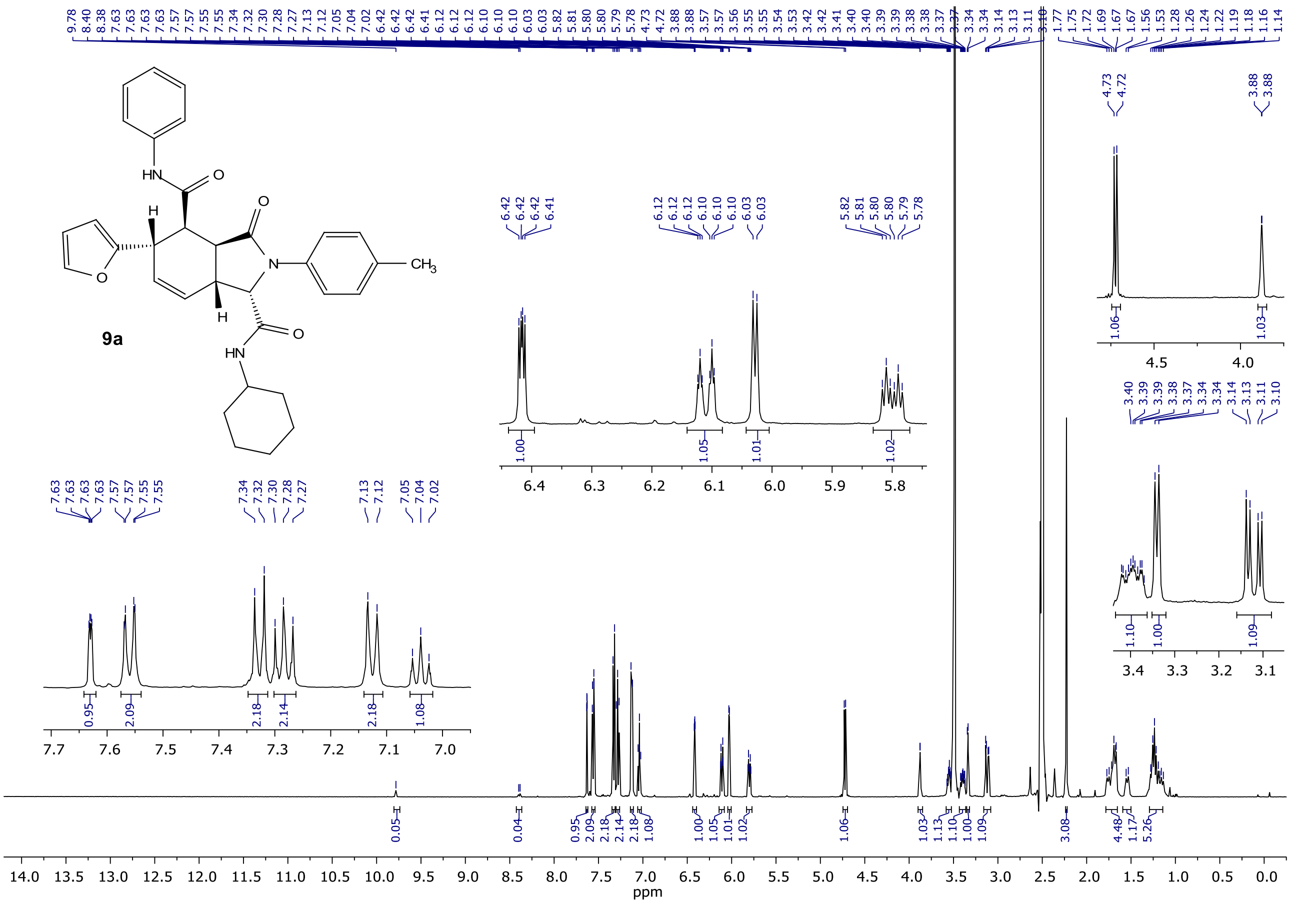
9a
ppm

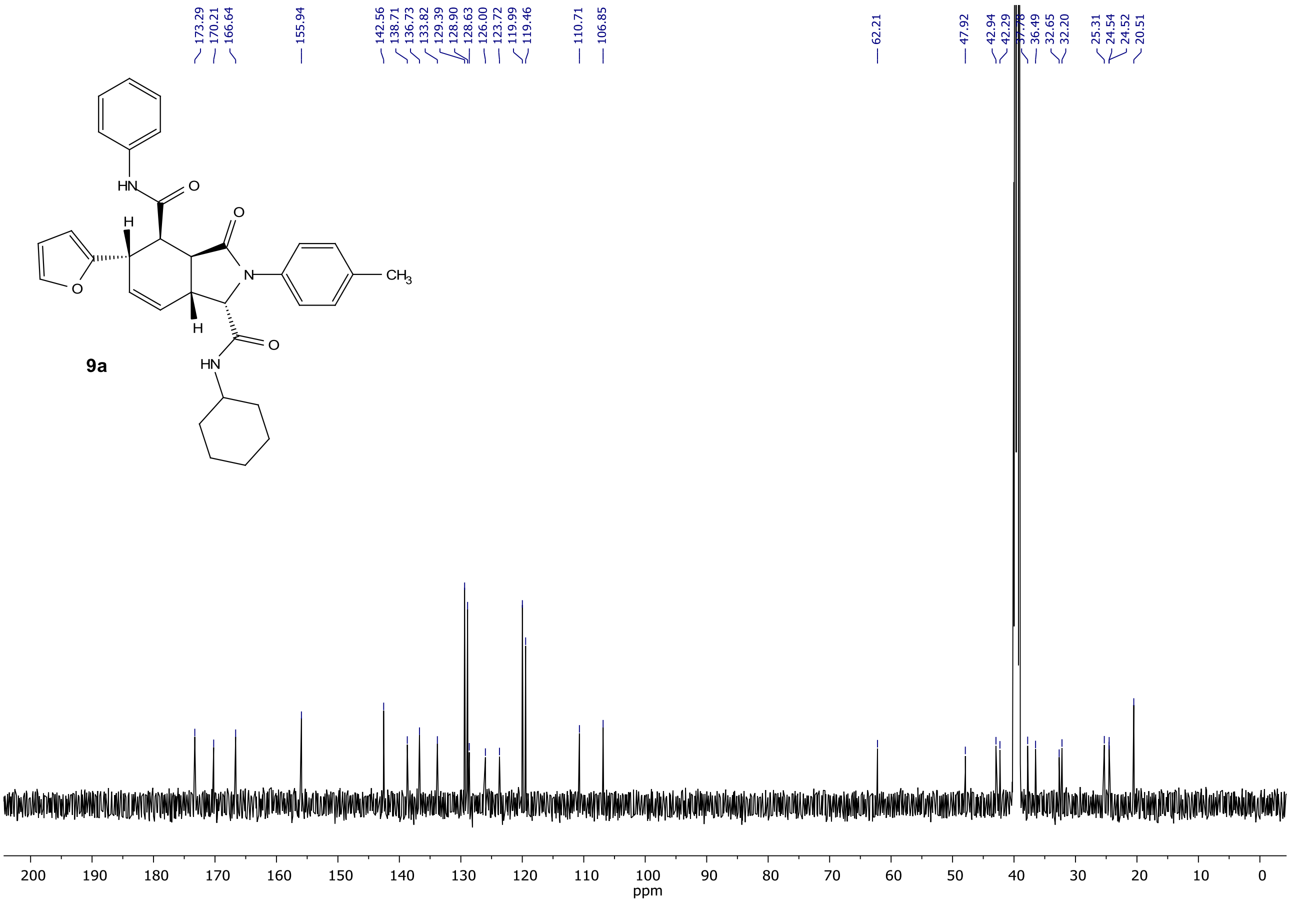
9a
ppm

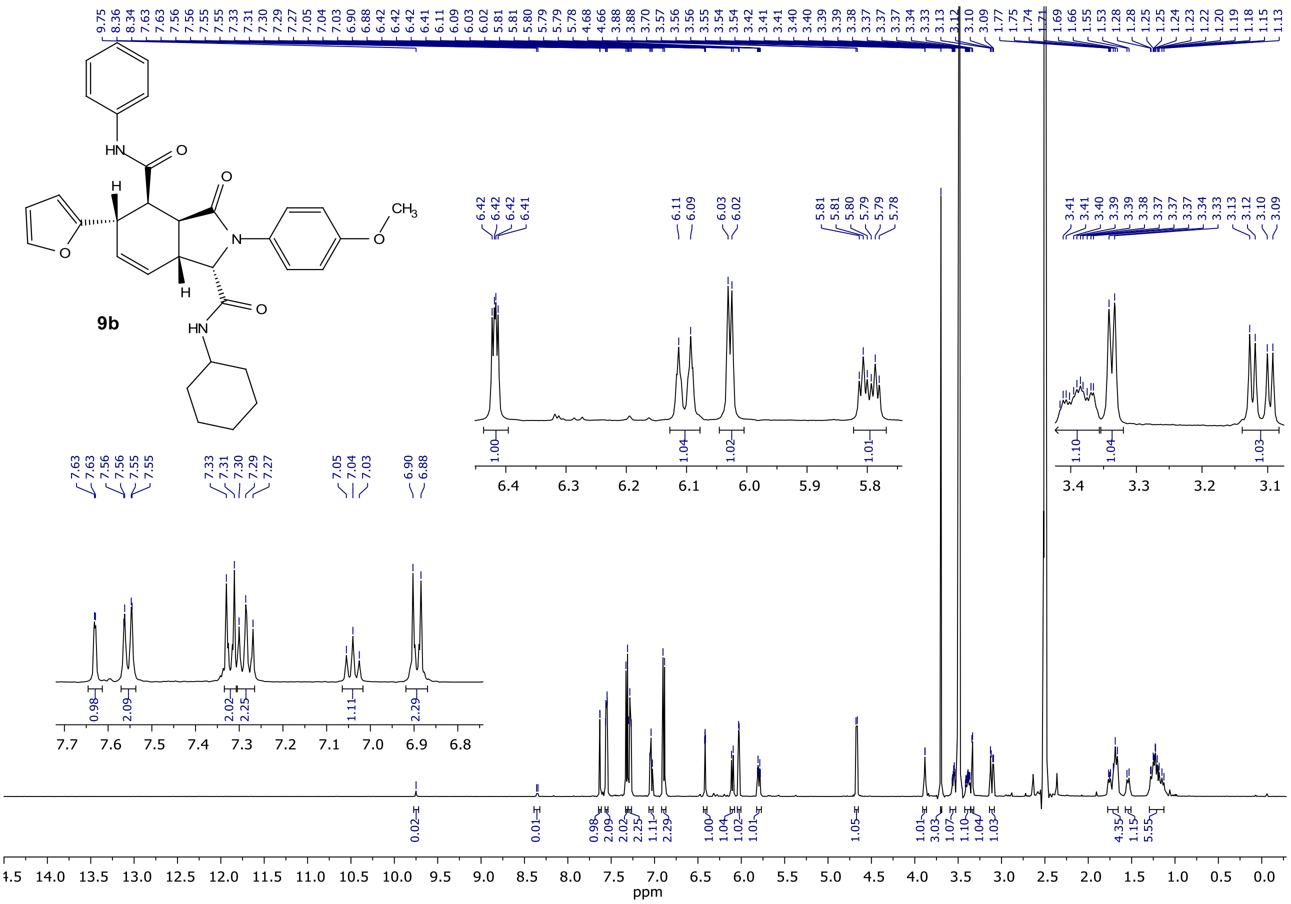
9b
HN
O
N
CH3
H
ppm

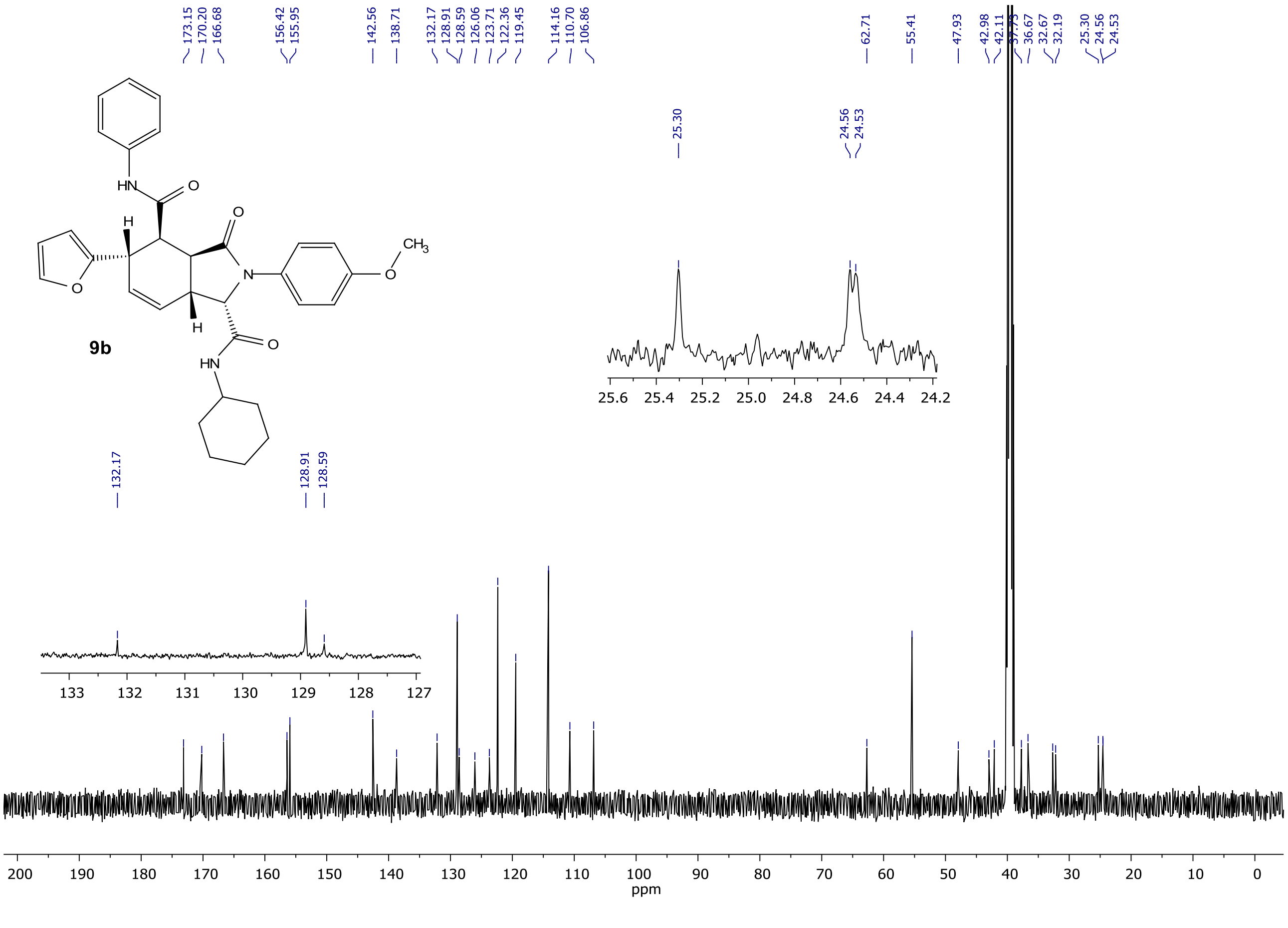
9b
HN
O
N
CH3
H
ppm
